# SNP heterozygosity, relatedness and inbreeding of whole genomes from the isolated population of the Faroe Islands

**DOI:** 10.1186/s12864-023-09763-x

**Published:** 2023-11-23

**Authors:** Hannes Gislason

**Affiliations:** Faculty of Science and Technology, University of the Faroe Islands, Tórshavn, Faroe Islands

**Keywords:** WG SNPs, Heterozygosity, Relatedness, *ROH*, Inbreeding, *maf*, Ancestry, Population isolate

## Abstract

**Background:**

The population of the Faroe Islands is an isolated population but very little is known about it from whole genome sequencing. The population of about 50000 people has a high incidence of rare diseases e.g., 1:300 for Primary Carnitine Deficiency. A screening programme was implemented, and eleven persons were also whole genome sequenced at *x*37 coverage for diagnostic purposes of those cases that were not affected by the known mutations. The purpose of our study is to utilize the high coverage data to explore the genomic variation and the ancestral history of the population. We study the SNP heterozygosity, the pairwise relatedness from kinship, the inbreeding from runs of homozygosity *ROH*, and we find the minor allele frequency distribution. We estimate the population ancestry and the timing of the founding event by using the whole genomes from eight consenting individuals.

**Results:**

We find the number of SNPs and the heterozygosity for the eight individual samples, and for merged samples, for which we also study the relatedness. We find close relatedness between the supposedly unrelated individuals. From *ROH*, we interpret the high relatedness as an ancient property of the isolated population. A bottleneck event is estimated starting between years $\sim 50-300$ with a maximum consanguineous population in year $\sim 600$ and similarly consanguineous between years $500-700$. The ancestry analysis shows the population descends from founders of $>99\%$ European and $<1\%$ Admixed American ancestry. A distinct clustering near the central European and British populations of the 1000 Genome Project is likely the result of the population isolation and genetic drift. The minor allele frequency distribution suggests many rare variants.

**Conclusions:**

The ancestry is mainly European while the inbreeding is higher compared to European populations and population isolates. The Faroese population has inbreeding more like ancient Europeans. We discovered a bottlenecked and consanguineous population event and estimated it starting in the 1st-4th century as compared to the oldest archaeological findings from the 4th-6th century.

**Supplementary Information:**

The online version contains supplementary material available at 10.1186/s12864-023-09763-x.

## Background

The population of the Faroe Islands is an isolated population [1–8]. The Viking settlement of the Islands was in the 9th century around year $825-875$ [2]. Presumably from Scandinavian, mainly Norse Vikings, but mixed with independent colonization, Viking intermarriages, and from bringing with them both female and male slaves from the British Isles [2]. An older settlement of Irish monks is believed to have occurred about year 650 which later deserted the Islands due to the appearance of the Vikings [2].

Even earlier settlement in the 4th century is proposed by archaeological evidence from carbon dating of barley grains placing human colonization in two pre-Viking phases within the 4th-6th and late 6th-8th centuries [9]. The first settlement in another study was estimated to year 500 (CI: 370-610) from an increase in fecal biomarker concentrations and by the first appearance of sheep DNA [10].

Since the settlement the population has been relatively isolated due to its remote geographical location. The population size was nearly constant at about 4000 for 500 years between $1320-1820$ until it increased more rapidly in 200 years to its modern population of about 50000 [7].

In isolated populations, the founder effect, genetic bottlenecks, and genetic drift have worked to increase the frequency of rare variants, leading to increased power to detect those variants in genome-wide studies, and genetic isolates have unique profiles for rare disease alleles [11]. Highly inbred populations have increased frequencies of homozygosity and decreased number of heterozygotes, and the high degree of inbreeding increases the incidence of recessive allele disorders [12].

In the Faroe Islands, the Primary Carnitine Deficiency (PCD) is a recessive allele disorder with a very high incidence of 1:300 and it may cause sudden death [13]. A screening programme for PCD and gene mutations were therefore used, respectively, to find and verify cases with carnitine levels below $7 \mu mol/l$ [13, 14]. Whole genome (WG) sequencing of patients without PCD mutations were also performed [14]. Subsequently, eleven genomes of six cases and five controls were stored in the Genetic Biobank of the Faroe Islands.

The demographic history, the population structure and the disease linkage studies of the Faroese population have previously mainly been performed with microsatellite and mitochondrial DNA markers [1–3] and more recently with genotype SNP arrays and whole exome sequencing [4–6, 8]. Therefore, very little is known about the Faroese population from WG sequencing.

A WG sequencing of the Faroese population was planned by local researchers and a scientific advisory board (FarGen SAB) that recommended analysing the eleven existing genomes in a statistical pre-project not focusing on disease [15]. After ethical approval, eight persons consented to the study, but computational access to the WGs was delayed for years.

Individual genomes ($n=1$) are interesting for personalized medicine, pairs of genomes ($n=2$) for relatedness and genetic counselling, and larger groups of genomes for case control studies and population genomics.

Sometimes small sample sizes are adequate even for population genomics. It is suggested that only a few samples of about $n=6-8$ are needed to obtain accurate population genetic parameters because the large number of markers in sequencing studies compensate for the small sample size [16]. The optimal design for heterozygosity may be the deep sequencing of a small number of individuals (e.g. $n=5-10$) from each population, rather than shallower sequencing of many individuals [17].

FarGen recruited 1541 participants [7] for exome sequencing [8] and will use WG sequencing focusing on population genomics and four diseases [18]. This will allow future WG studies with larger sample sizes from the Faroese population.

The purpose of our study is to utilize the previously generated high coverage data to explore the WG variation and demographic history of the population. In particular, we assess the contribution of historical bottlenecks and small population size in generating within-sample homozygosity and between-sample relatedness, with the expectation that their contribution should be high. We study the SNP heterozygosity, the pairwise relatedness from kinship, the inbreeding from runs of homozygosity *ROH*, and we find the minor allele frequency distribution. We infer the population ancestry, and we search for bottleneck effects by using the whole genomes from eight consenting individuals. Finally, we estimate the timing of the founder event and compare it with the dating of the oldest archaeological findings from the Faroe Islands.

## Results

We present the first bioinformatic analysis of the WGs to infer the genetic diversity of eight individual and merged genomes for both basic (Additional files 1, 2, 3 and 4) and advanced filtering criteria (Additional files 5, 6, 7 and 8). Unless otherwise specified, when we refer to numbers, figures, and tables, it is for one of the criteria (the basic), since most of our analysis is not much affected by the filtering. All the analysis for both filtering criteria is shown in our tables and in the supplementary material (Additional files 1, 2, 3, 4, 5, 6, 7, 8, 9, 10 and 11).

The $N_{SNP}$, missing rate and *H* for the individual samples and summary statistics are given in the supplementary material. The missing rate is low (0.0004) for all the samples. The median (*iqr*) $N_{SNP}=3559354$ (17756) and the median (*iqr*) $H=0.600$ (0.004) (Table 1, Additional file 1: Tables S1.1-1.4).

The variability in $N_{SNP}$ and *H* between the individual samples is very small. The maximum and minimum $N_{SNP}$ are 0.5 and $2\%$ from the median, respectively. The maximum and minimum *H* are 1 and $4\%$ from the median, respectively.

The $N_{SNP}=3559354$ for individual samples is within $1\%$ from the 3.60M SNPs per sample in the 1000 Genomes Project (1000GP) [19].

For the merged samples the missing rate is high ($\approx 0.49-0.51$, Additional file 1: Table S1.5), while both $N_{SNP}$ and *H* are like the values for individual samples (Additional file 1: Table S1.2). The high missing rate is because the merging of samples marks the SNPs as missing that are not shared in all the samples.

After missing genotype filtering of the merged samples, the missing rate is 0, $N_{SNP}=1136546$ SNPs shared by all the samples and the median (*iqr*) $H=0.292$ (0.011) (Table 1, Additional file 1: Tables S1.6-1.8).

The number of SNPs used to calculate the pairwise *R* is the number of SNPs shared between the two samples in each of the 28 possible relationships. Before the missing genotype filtering, the median (*iqr*) $R=0.669$ (0.010) is based on 28 different $N_{SNP}$ with the median (*iqr*) $N_{SNP}= 2348854$ (22700) (Additional file 1: Tables S1.9-1.10). After the filtering, the median (*iqr*) $R=0.515$ (0.016) is based on the common $N_{SNP}=1136546$ shared by all the samples (Table 1, Additional file 1: Tables S1.9-1.10).

The lower *H* (Table 1) for the merged samples after missing genotype filtering (Additional file 1: Table S1.8) suggests that more heterozygous than homozygous genotypes are being filtered out. This is confirmed by the KING quality report (--bySNP) for the merged sample without the filter (Additional file 1: Table S1.11). The overall heterozygosity $H=0.597$ across all 7080960 SNPs in the merged sample without the filter (Additional file 1: Table S1.12) equals the mean $H=0.597$ across the merged samples without the filter (Additional file 1: Table S1.8), and the mean $H=0.597$ across single samples (Table 1, Additional file 1: Table S1.4). However, *H* is highest for the 1724918 SNPs only called in one sample and lowest ($H=0.291$) for the 1136546 SNPs called in all samples (Additional file 1: Table S1.11).

Since the SNPs not found in all of the samples are marked as missing, there will in total be more heterozygous (14325880) than homozygous (4989123) genotypes marked as missing in the merged sample for call rates between $0.125-0.875$ (Additional file 1: Table S1.11). This decreases the mean heterozygosity from $H=0.597$ across all 7080960 SNPs, to 0.291 for 1136546 SNPs when we filter out the missing genotypes from the merged sample (Additional file 1: Tables S1.11-1.12).

**Fig. 1:**
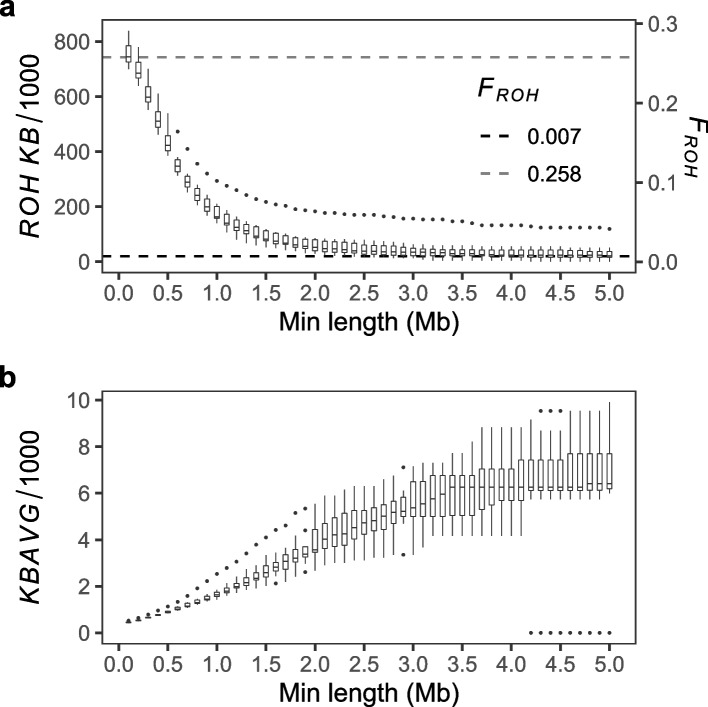
*ROH* of the eight merged genomes (autosomes) from the Faroe Islands. **a, b** Total $ROH=KB/1000$ and average $ROH=KBAVG/1000$ are the total and average length of *ROH*, respectively, in the PLINK --homozyg report for variable minimum segment length --homozyg-kb. This we varied from 100 to 5000kb in 100kb increments. **a** Boxplots of the total *ROH* (left axis) and of the inbreeding coefficient $F_{ROH} = KB/L$ (right axis) in which $L = 2881033.286$kb is the autosome length. The median inbreeding $F_{ROH>5}=0.007$ (black dashed line) is the lowest level of recent inbreeding, which is like the average pedigree inbreeding of 0.0067 and 0.0081 estimated for multiple scelerosis patients and controls, respectively, from the Faroese population [23]. The $F_{ROH>0.1}=0.258$ (gray dashed line) is the highest level of both recent and ancient inbreeding, which is like $F_{ROH>0.1}$ for the European population of the 1000GP [24]. At 1.5Mb minimum length, $F_{ROH>1.5} =0.029$ is like inbreeding for ancient genomes of simple and early complex agriculturalists in West and Central Eurasia, respectively [25]. This inbreeding is higher compared to $F_{ROH>1.5}=0.0039$ and 0.0156 for present-day genomes from West and Central Eurasia in the Human Genome Diversity panel [25]. It is also higher than $F_{ROH>1.5}$ of 0.013 and 0.011 for the contemporary population isolates of the endogamous Dalmatians in Croatia and the endogamous Orcadians in Orkney, respectively [20]

In our study, *R* (Table 1) is unexpectedly high for the reported unrelated participants (Bjarni á Steig, personal communications, October 8, 2019). To explain the high *R* derived from kinship we analysed *ROH* [20–22] of eight merged samples for different minimum *ROH* lengths of $0.1-5$Mb (Fig. 1). We find the *ROH* median (*iqr*) (Table 2, Additional file 2: Tables S2.2-2.6):

- number of segments *NROH* from 1655.5 (92.2) down to 3.5 (3.5).
- total lengths *SROH* from 744.3 (59.4) down to 21.5 (21.2) Mb.
- average lengths *AVROH* from 0.45 (0.03) up to 6.41 (1.50) Mb.
- inbreeding from $F_{ROH>0.1}$ = 0.258 (0.021) down to $F_{ROH>5}$ = 0.007 (0.007).
- relatedness from $R_{ROH>0.1}$ = 0.517 (0.041) down to $R_{ROH>5}$ = 0.015 (0.015).

The corresponding statistics for the advanced filtering criteria are in general similar or somewhat higher (Table 2, Additional file 6: Tables S6.2-6.6).

For the 1.5Mb minimum length commonly used in inbreeding studies [20, 25–27], we find the median (*iqr*) $NROH= 33.5$ (4.8), $SROH= 82.3$ (36.1) Mb, $AVROH= 2.59$ (0.48) Mb and $F_{ROH>1.5}=0.029$ (0.013) (Table 2).

For comparison, a large Alzheimer’s disease study of $n=21190$ unrelated Europeans found much smaller median values $NROH= 14.0$, $SROH= 28.1$Mb, $AVROH= 1.95$Mb and $F_{ROH>1.5}=0.009$ [27]. The inbreeding $F_{ROH>1.5}$ in our study for both our basic and advanced filtering is in general higher as compared with European populations and population isolates (Table 3).

Next, we compare *R* from kinship with $R_{ROH>x}$ from *ROHs* and find them similar for minimum *ROH* lengths $x=0.1-0.2$Mb. For the basic filtering, the median $R=0.515$ is most like the median $R_{ROH>0.1}= 0.517$, and for the advanced filtering, the median $R=0.481$ is most like the median $R_{ROH>0.2}=0.485$. The discrepancies between the medians are $<1\%$ and the means are nearly equal (Table 1). This suggests the evolutionary origin of the high *R*.

$F_{ROH>0.1}$ captures both ancient and recent inbreeding. The $F_{ROH>0.1}$ in our study is like $F_{ROH>0.1}$ for the European population of the 1000GP [24].

$F_{ROH>5}$ only captures recent inbreeding, because $F_{ROH>5}$ compares with pedigree inbreeding [20]. Indeed, $F_{ROH>5}=0.007$ (0.007) in our study (Table 2) is like the pedigree inbreeding of 0.0067 estimated for 58 multiple sclerosis patients and 0.0081 for 10 controls from the Faroese population [23].

**Fig. 2:**
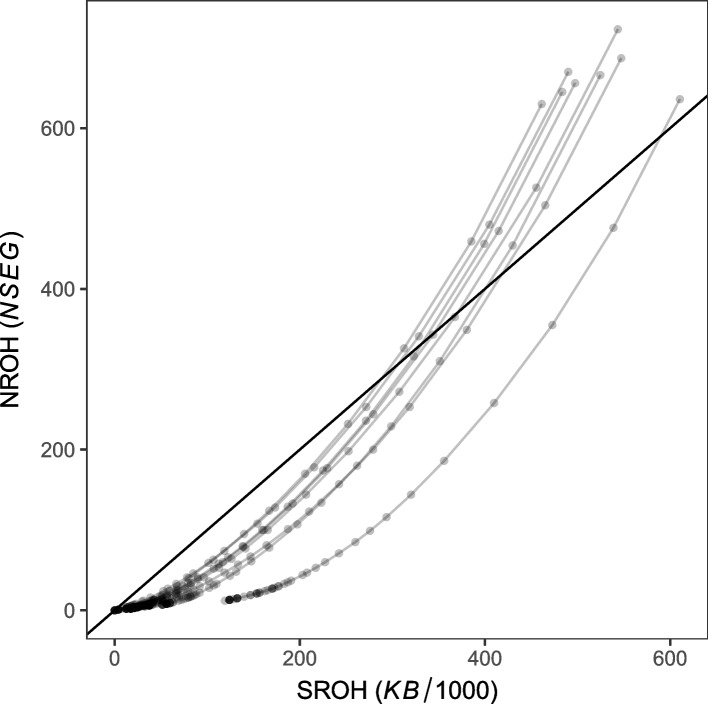
Plots for each sample of the number of runs of homozygosity NROH (*NSEQ*) against the total length of runs SROH (*KB*/1000) shown for variable minimum *ROH* lengths $0.4-5$Mb (transparent gray points, to show overplotting as darker points). The eight gray lines connecting the points show the nonlinear trajectories travelled by each sample from above the diagonal (black line) for the smallest minimum *ROH* lengths, crossing below the diagonal for increasing minimum lengths, and finally approaching towards the diagonal for the longest minimum *ROH* lengths

Plotting the number of *ROH* against the total length of *ROH* [22] for our data (Fig. 2), suggests a bottlenecked and consanguineous population for minimum *ROH* lengths above 0.6Mb (Additional file 2: Figs. S2.1-2.2). We use the number of *ROH* against the total length of *ROH* for each sample to infer the minimum *ROH* lengths at the start and at the maximum, respectively, of the bottlenecked and consanguineous population effect (Methods, Additional file 2: Figs. S2.3-2.4).

**Fig. 3:**
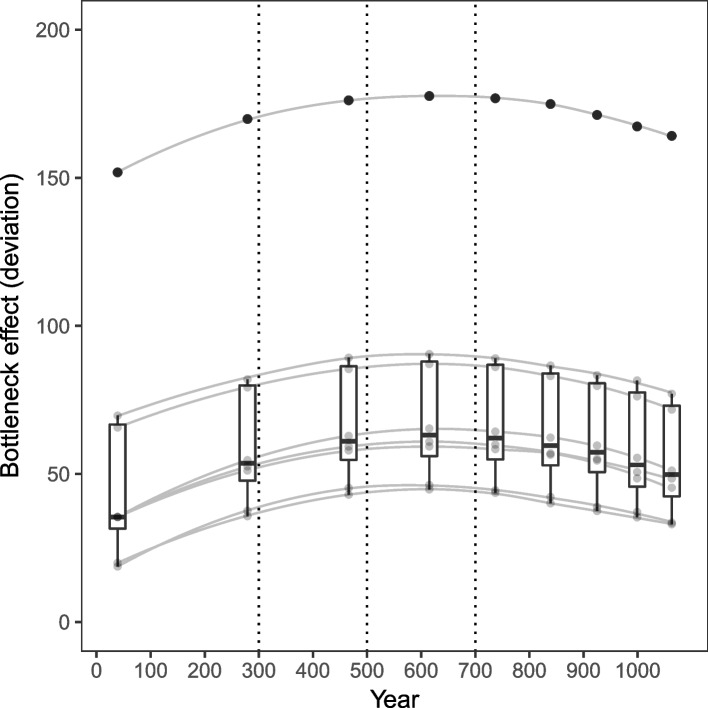
Plot of the deviations for each sample from the linear diagonal in the NROH versus SROH plots shown for minimum *ROH* lengths $0.7-1.5$Mb that are transformed to the corresponding estimated time in years. The boxplots with overlaid points (transparent gray) and boxplot outliers (black) show the median deviation and the variability of the deviation. The sample trajectories (smoothed gray lines) show the development through time of the deviation for each sample. The maximum deviation for all samples is found at about 1.0Mb corresponding to year 615. The bottleneck and consanguineous population event is estimated starting at year $\sim 50-300$ and increasing with a maximum consanguineous population in year $\sim 600$ and similarly consanguineous between years $500-700$. Thereafter, the bottleneck effect slowly decreases. For comparison the three dotted lines (black) illustrate the dating of the oldest archaeological findings from the Faroe Islands: two pre-Viking colonization events at about years $300-500$ and years $500-700$ [9], and the first apperance of sheep DNA in year 500 (CI: $370-610$) [10]

We convert the *ROH* lengths to estimates of the timing of the events (Methods, Additional file 2: Figs. S2.5-2.6). Finally, we plot the events on the estimated time scale and estimate the start of the bottleneck and consanguineous event to between years $40-280$ or $\sim 50-300$, and a maximum consanguineous population effect at year 615 or $\sim 600$ and similarly high between years $\sim 500-700$ (Fig. 3, Additional file 2: Figs. S2.7-2.9).

The sample size is too low to accurately estimate the *maf* spectrum. However, $maf=0, 0.0625$ for $33.4, 13\%$ of the variants, respectively, indicating many rare variants having $33.4\%$ with $maf<0.0625$ (Additional file 3: Table S3.2, Fig. S3.1).

The MDS inferred ancestry of the samples is $>99\%$ European with the remaining $<1\%$ being Admixed American (Additional file 4: Table S4.2). The Faroese WGs cluster near to the central European and British populations of the 1000GP (Additional file 4: Figs. S4.1 and S4.2). With precaution for our small sample this is a more distinct clustering than for Faroese exomes [6]. Earlier studies have emphasized genetic drift [1, 3], and the distinct clustering in our study is likely the result of the population isolation and genetic drift.

**Table 1:** Number of SNPs $N_{SNP}$, heterozygosity *H* and relatedness *R* for the eight genomes (autosomes) from the Faroe Islands. $N_{SNP}$, *H* and *R* were derived from the KING --bysample and --kinship methods, respectively (Additional files 1 and 5 for our basic and advanced filtering, respectively). We also compare *R* from kinship with $R_{ROH>x}$ from *ROHs* (Methods, Additional files 2 and 6 for our basic and advanced filtering, respectively). The median and mean number of $N_{SNP}$, *H*, *R*, $R_{ROH>0.1}$ and $R_{ROH>0.2}$ are shown for both filtering criteria used before further processing with PLINK and KING. The $N_{SNP}$, *H* are shown for the eight individual and merged samples, and *R*, $R_{ROH>x}$ are shown for the merged samples. For the basic filtering *R* is like $R_{ROH>0.1}$ and for the advanced filtering *R* is like $R_{ROH>0.2}$

Parameter	*med*	*iqr*	*mean*	*sd*	samples
**Filter**	$\boldsymbol Q\boldsymbol U\boldsymbol A\boldsymbol L\boldsymbol>\mathbf{30}$				
$N_{SNP}$	3559354	17756	3552025	26065	individual
	1136546	0	1136546	0	merged
*H*	0.600	0.004	0.597	0.010	individual
	0.292	0.011	0.291	0.007	merged
*R*	0.515	0.016	0.524	0.042	merged
$R_{ROH>0.1}$	0.517	0.041	0.525	0.031	merged
$R_{ROH>0.2}$	0.475	0.040	0.483	0.031	merged
**Filter**	***PASS*,** $\boldsymbol Q\boldsymbol U\boldsymbol A\boldsymbol L\boldsymbol>\mathbf{30}\boldsymbol,\boldsymbol\;$ $\boldsymbol F\boldsymbol M\boldsymbol T\boldsymbol/\boldsymbol D\boldsymbol P\boldsymbol U\boldsymbol>\mathbf{10}$				
$N_{SNP}$	3243489	15740	3234556	29368	individual
	1002681	0	1002681	0	merged
*H*	0.607	0.004	0.605	0.010	individual
	0.282	0.011	0.281	0.007	merged
*R*	0.481	0.017	0.491	0.045	merged
$R_{ROH>0.1}$	0.519	0.043	0.524	0.030	merged
$R_{ROH>0.2}$	0.485	0.044	0.491	0.031	merged

**Table 2:** The *ROH* statistics for the number of segments *NROH*, total lengths *SROH*, average lengths *AVROH*, inbreeding $F_{ROH}$, relatedness $R_{ROH}$ for the eight genomes (autosomes) from the Faroe Islands at the subset 0.1, 1.5, 5.0Mb of the minimum *ROH* lengths. The *ROH* statistics are shown for both the basic and advanced filtering criteria (for more summary statistics, see Additional files 2 and 6)

Parameter	Mb	*med*	*iqr*	*mean*	*sd*
**Filter**	$\boldsymbol Q\boldsymbol U\boldsymbol A\boldsymbol L\boldsymbol>\mathbf{30}$				
*NROH*	0.1	1655.5	92.2	1666.1	55.2
	1.5	33.5	4.8	35.5	10.3
	5.0	3.5	3.5	4.4	3.7
*SROH*	0.1	744.3	59.4	756.0	44.9
	1.5	82.3	36.1	101.1	51.6
	5.0	21.5	21.2	34.5	37.3
*AVROH*	0.1	0.45	0.03	0.45	0.03
	1.5	2.59	0.48	2.77	0.66
	5.0	6.41	1.50	6.38	2.92
$F_{ROH}$	0.1	0.258	0.021	0.262	0.016
	1.5	0.029	0.013	0.035	0.018
	5.0	0.007	0.007	0.012	0.013
$R_{ROH}$	0.1	0.517	0.041	0.525	0.031
	1.5	0.057	0.025	0.070	0.036
	5.0	0.015	0.015	0.024	0.026
**Filter**	***PASS*,** $\boldsymbol Q\boldsymbol U\boldsymbol A\boldsymbol L\boldsymbol>\mathbf{30}\boldsymbol,\boldsymbol\;$ $\boldsymbol F\boldsymbol M\boldsymbol T\boldsymbol/\boldsymbol D\boldsymbol P\boldsymbol U\boldsymbol>\mathbf{10}$				
*NROH*	0.1	1513.5	53.2	1520.0	51.4
	1.5	40.0	7.0	40.0	7.5
	5.0	4.5	4.2	5.0	3.3
*SROH*	0.1	747.8	62.0	754.8	43.5
	1.5	104.5	49.1	120.3	54.9
	5.0	30.2	37.0	46.5	46.8
*AVROH*	0.1	0.49	0.03	0.50	0.04
	1.5	2.68	0.39	2.93	0.89
	5.0	7.65	1.17	8.17	2.45
$F_{ROH}$	0.1	0.260	0.022	0.262	0.015
	1.5	0.036	0.017	0.042	0.019
	5.0	0.010	0.013	0.016	0.016
$R_{ROH}$	0.1	0.519	0.043	0.524	0.030
	1.5	0.073	0.034	0.084	0.038
	5.0	0.021	0.026	0.032	0.032

**Table 3:** Inbreeding $F_{ROH>1.5}$ and *SROH*, the total sum of *ROH* in modern and ancient European populations for $ROHs>1.5$Mb. The data for the Faroe Islands are from the present study in which: (bas., PLINK default), (adv., PLINK default) refers to our basic and advanced filtering, respectively, and the default *ROH* parameters used in PLINK except for the minimum *ROH* length (Additional files 2 and 6). The (bas., PLINK [20]), (adv., PLINK [20]) refers to our basic and advanced filtering, respectively, and the same PLINK parameters used in the reference [20] (Additional files 9 and 10). The *SROH* are mean values rounded to integers and the remaining numbers are rounded to 3 decimal digits

Population/Region	${\boldsymbol F}_{\mathbf R\mathbf O\mathbf H\boldsymbol>\mathbf1\boldsymbol.\mathbf5}$				*SROH*	Ref.
	*med*	*iqr*	*mean*	*sd*	*Mb*	
Faroe Islands (adv., PLINK default)	0.036	0.017	0.042	0.019	120	HG
Faroe Islands (bas., PLINK default)	0.029	0.013	0.035	0.018	101	HG
Faroe Islands (adv., PLINK [20])	0.018	0.011	0.023	0.015	67	HG
Faroe Islands (bas., PLINK [20])	0.018	0.010	0.022	0.014	65	HG
Endogamous Dalmatians			0.013		35	[20]
Endogamous Orcadians			0.011		28	[20]
Croatians			0.007		18	[20]
Mixed Dalmatians			0.006		15	[20]
Mixed Orcadians			0.005		14	[20]
CEU			0.003		8	[20]
Scottish			0.003		7	[20]
Half Orcadians			0.002		6	[20]
CEU			0.004	0.002	13	[26]
FIN			0.009	0.004	26	[26]
GBR			0.006	0.004	17	[26]
IBS			0.006	0.005	18	[26]
TSI			0.005	0.003	13	[26]
Hunter-gatherers	0.063	0.026				[25]
Simple agriculturalists	0.029	0.014				[25]
- West Eurasia	0.029	0.012				[25]
- Central Eurasia	0.020	0.017				[25]
Early comp. agriculturalists	0.025	0.011				[25]
- West Eurasia	0.024	0.010				[25]
- Central Eurasia	0.027	0.011				[25]
Adv. comp. agriculturalists	0.016	0.009				[25]
- West Eurasia	0.021	0.006				[25]
- Central Eurasia	0.015	0.006				[25]
Human Genome Div. Panel	0.007	0.022				[25]
- West Eurasia	0.004	0.005				[25]
- Central Eurasia	0.016	0.034				[25]
European ancestry	0.009		0.011	0.007	32	[27]

## Discussion

We studied the Faroese population using individual and merged samples to explore the WG variation and demographic history of the population.

For $N_{SNP}$ and *H* of the individual samples, the maximum of $2-4\%$ difference in sampling a single individual as compared to the median for the eight individuals, suggest small effects of the sample size for samples that are not merged.

When the samples are merged, the missing rate increases, since the SNPs not called in all of the samples are marked as missing. Before filtering out all the missing genotypes from the merged sample, the $N_{SNP}$ and *H* are like for the individual samples. Here the *R* was based on 28 different $N_{SNP}$ shared by only the two samples of each relationship. After filtering out the missing genotypes, the $N_{SNP}$ decreases to the smaller number of SNPs shared in all the samples being merged, and both *H* and *R* decreases.

We showed that for the merged sample, more heterozygous than homozygous genotypes were marked as missing for call rates between $0.125-0.875$, i.e. called in one to seven of the eight samples. The *H* was highest for those SNPs only called in one sample and lowest for the SNPs called in all the samples. Therefore, *H* decreased when the missing genotypes were filtered out.

The reason for *H* being highest for those SNPs only called in one sample and lowest for the SNPs called in all of the samples is maybe that the private SNPs are of more recent origin and tend to be heterozygous. Also, possibly some private genotyping errors occur that are not shared between all the samples.

An SNP homozygosity of 0.6905 (i.e. $H=0.310$) for 28 controls and 0.6918 (i.e. $H=0.308$) for 29 cases of multiple sclerosis was found in the Faroe Islands [4]. This is close to (6% from) our $H=0.292$ for $n=8$. An earlier study with 14 classical markers found Iceland to have the lowest $H=0.424$, as compared to the genetic variation of 10 European populations, including the Faroes with $H=0.431$ [28].

A low genetic variation is often associated with low fitness, inbreeding and disease [20, 21]. However, for identical markers and two groups of samples, the larger sample tends to have smaller *H* [29]. Similarly, perhaps the higher number of 29 cases versus 28 controls [4] caused the lower *H* (i.e. the slightly higher homozygosity for cases).

Estimates of *H* based on polymorphic markers only, (i.e. SNP heterozygosity) are biased by sample size for samples run together in a single Stacks run, as opposed to when they are run individually, with smaller samples producing larger estimates [17]. Without such bias, the consistency of *H* estimates for small subsamples of different sizes [16] is due to these subsamples not being refiltered by polymorphism [17].

The study [17] suggests that this effect of the sample size can be avoided by basing the *H* estimates on the genome-wide sequence without any minor allele frequency filtering, thus including both the monomorphic as well as the polymorphic sites, and by filtering out all the missing genotypes [17].

As recommended [17] we did not filter on the minor allele frequency, and we filtered out the missing genotypes. In agreement with [17], we showed that $N_{SNP}$ and *H* were not much variable by sample size for samples that were not merged. This is because the maximum of $2-4\%$ difference in sampling a single individual as compared to the median for the eight individuals showed small effects of the sample size for samples that are not merged. The merging itself did not significantly change $N_{SNP}$ and *H*, but only after filtering out the missing genotypes from the merged sample, $N_{SNP}$ and *H* decreased. Our results after merging and missing genotype filtering cannot be compared with [17], which used filtering of subsamples of different sample sizes but analysed the samples individually without any merging.

We did our own subsampling study (not presented) which showed diminishing decreases of *H* with increasing merge size for $n=2-8$, even for the filtering recommendations [17]. A larger merge size than 8 is needed to establish when this effect vanishes. For $n>5$ we observed a nearly linear decrease of *H* of about $1\%$ for each increase in *n*: $7\%$ at $n=6$, $6\%$ at $n=7$, $4.6\%$ at $n=8$. If a linear decrease of about $1\%$ for each increase in *n* continues for $n>8$, the *H* of $0.282-0.292$ for merged samples in our study (Table 1) will decrease further and settle at about $0.25-0.26$ for $n>12$.

With respect to *R*, several estimators are biased by sample size [30]. However, the king --kinship method performs pair-wise relationship inference using only information from the two individuals under comparison and, the inference is claimed invariant to inclusion of any additional samples and to use of different SNP panels [31]. The SNP panel changes in our study because the number of SNPs decrease for the merged samples after missing genotype filtering, but different SNP panels should not affect *R* [31]. Therefore, it was unexpected that the estimates of *R* decreased for the merged sample after missing genotype filtering.

However, the decrease of *R* is likely from the fact that before the missing genotype filtering of the merged sample, the *R* estimates are based on the number of SNPs shared only by the two samples of the relationship, while after the missing genotype filtering, the *R* estimates are based on the (equal) number of SNPs shared by all the samples. In both cases *R* was much higher than expected for the reported unrelated participants of the study, and highest (0.669) before and lowest (0.515) after the missing genotype filtering.

With respect to *ROH*, the *ROH* procedures can distinguish between evolutionary and familial relatedness, while SNP-by-SNP estimators cannot [24]. WG sequencing with coverage $>x30$ is best for finding *ROHs* of any size because genotype calling is robust for low minor allele frequency *maf* [32].

With default PLINK parameters, the $F_{ROH>1.5}=0.029$ (0.013) and 0.036 (0.017) (Table 3) for the basic and advanced filtering criteria, respectively, seem higher than the previously reported inbreeding for the Faroese population $F_{ROH}=0.015\pm 0.001$ (no minimum length stated, probably default $F_{ROH>1}$) [6]. It is also higher than the inbreeding $f=0.018$ based on observed and expected number of homozygotes in each individual [4]. Both studies [4, 6] used *maf* filtering and *LD* pruning. The inbreeding $F_{ROH>1.5}=0.029$ is like ($6\%$ below) the expected value $\theta$ (coancestry of parents) for half-cousin relationship $\theta =E[\theta ^{'}]=0.031$, where $\theta ^{'}$ is the realized genomic regions shared identity-by-decent [33]. The $F_{ROH>1.5}=0.036$ (0.017) is $16\%$ higher.

The $F_{ROH>1.5}=0.029$ compares with $F_{ROH>1.5}=0.029$ (0.012) and 0.027 (0.011) for ancient genomes of simple and early complex agriculturalists in West and Central Eurasia (Table 3). This is higher compared to $F_{ROH>1.5}=0.004$ (0.005) and 0.016 (0.034) for present-day genomes from West and Central Eurasia in the Human Genome Diversity panel, respectively, with median $F_{ROH>1.5}=0.007$ (0.022) (Table 3) suggesting that human inbreeding has decreased [25].

The $F_{ROH>1.5}$ in our study is also high compared to contemporary population isolates (Table 3). For example, the endogamous Dalmatians in Croatia have a mean $F_{ROH>1.5}$ of 0.013, and the endogamous Orcadians in Orkney 0.011, while Croatians have 0.007 and the Scottish 0.003 [20].

The study [20] used similar conditions to ours, with no *maf* filtering or *LD* pruning and mainly default PLINK parameters. Except for varying the minimum *ROH* length between 0.5, 1.5 and 5Mb, using a maximum gap between two consecutive homozygous SNPs of 100kb and a *ROH* was called if it had a minimum of 25 SNPs. We mainly used the default PLINK parameters with the gap of 1000kb and the minimum 100 SNPs to call a *ROH*. Except for when comparing our results to European populations and population isolates using the same PLINK parameters as in [20].

*ROH* results are sensitive to both the SNP set and the PLINK parameters. The lack of consensus conditions complicates the comparison between studies [34–36]. Reducing our gap to 100kb and using the minimum of 25 SNPs to call a *ROH* reduces the median and mean $F_{ROH>1.5}$ to 0.018 ($iqr=0.010$) and 0.022 ($sd=0.014$), respectively, and with these parameters $F_{ROH>1.5}$ is nearly identical for both the basic and advanced filtering criteria (Table 3, Additional files 9 and 10). This is still high compared to the isolated populations in the study [20] and more like the previously reported inbreeding of $f=0.018$ for the Faroese population [4]. It is also similar to the inbreeding for ancient genomes from simple agriculturalist in Central Eurasia or from advanced complex agriculturalists [25]. Similarly, with the 100kb gap and the minimum of 25 SNPs, the median $R_{ROH>0.1}$ is reduced to 0.472 (0.043). This is still relatively close to (8% from) $R=0.515$ derived from kinship.

The point we are making, is not to calibrate or fit *R* to $R_{ROH>x}$ by tuning *x*, but to show that the solution to $R = 2F_{ROH>x}$ is a small value of *x* for the observed high value of *R* found with KING in our study. For $x = 0.1-0.2$Mb for our basic and advanced filtering criteria, respectively, *R* is about equal to $2F_{ROH>x}$ (Table 1) supporting the hypothesis that the high *R* found with KING has an evolutionary origin. If, on the other hand, the solution had been $x > 5-10$Mb this would have suggested that the individuals were more recently related.

The *ROH* statistics in our study suggest a bottlenecked and consanguineous population having many short and some long *ROH* segments of ancient and recent origin, respectively. This is because bottlenecks introduce many short *ROH*, while consanguinity adds a small number of long *ROH* [22, 24]. Our plots of the number of *ROH* against the total length of *ROH* showed the development trough time of a bottlenecked and consanguineous population, with all points for the eight samples right shifted below the diagonal for minimum *ROH* lengths larger than 0.6Mb.

This was expected because the Faroe Islands are believed to be an isolated population with high levels of consanguineous marriages [4]. The population has likely experienced several bottlenecks followed by population expansion and genetic drift [3, 8]. A study of gene diversity at 15 unlinked microsatellite markers did not, however, find any sign of a severe bottleneck to have occurred within the approximately 1200 years’ history of the Faroese population, and instead, high *LD* primarily caused by random genetic drift [1].

From our *ROH* analysis, we identified a bottlenecked and consanguineous population event that we estimated started between years $\sim 50-300$ in the 1st-4th century, developing into a maximum consanguineous population in year $\sim 600$ in the 7th century and being similarly consanguineous during years $\sim 500-700$ in the 6th-8th century.

These estimates agree reasonably well with the oldest archaeological findings of human colonization in the Faroe Islands dated to two pre-Viking phases within the 4th-6th and late 6th-8th centuries [9]. The colonization of the Faroe Islands was estimated to year 500 (CI: $370-610$) in another study [10] with the confidence interval stretching between the 4th-7th century.

Our estimates are independent results that support the oldest archaeological findings of colonization dating within the 4th century, while suggesting that the colonization may have started even earlier in the 1st-4th century. In our study, the consanguineous effect is increasing between the 4th-6th century, highest between the 6th-8th century, whereafter it slowly declines. This seems consistent with the two pre-Viking phases within the 4th-6th and late 6th-8th centuries [9]. However, such an agreement could be accidental, and we should be aware of the danger of confirmation bias.

Ideally, the applied methodology should have been tested or calibrated with control samples from other isolated populations by inferring their bottlenecks and timing of colonization. For now, the dating’s of the oldest archaeological findings from the Faroe Islands [9] do serve as a single control of the applied methodology to eight sample replicates. With time, perhaps even older archaeological findings may be discovered in the Faroe Islands supporting our estimate of an even earlier colonization to the 1st-4th century. The start of the colonization is, however, challenging to estimate from *ROHs* because the observed consanguineous effect in *ROHs* do not necessarily imply the colonization of the Faroe Islands, but may have started before the colonization event.

## Conclusion

We analysed SNPs from eight individual genomes, and studied *ROHs*, *maf*, population structure inference, and ancestry for the eight merged genomes. The genomes were previously sequenced with *x*37 coverage in a clinical study in the Faroe Islands. The observed decrease of $N_{SNP}$, *H* and *R* for the merged sample illustrates the differences between individual and merged genomes. They probe the statistical properties of individuals, of pairwise relationships and ultimately of the population. Knowing these differences is vital for interpretation of genome-wide SNP case-control and population studies. These statistics are previously unknown for the Faroese population. These results should be further investigated for larger random samples. This will also improve the *maf* spectra indicating many rare variants having $33.4\%$ with $maf<0.0625$. The study participants were reported to be unrelated. From SNP kinship for eight merged genomes, and with our basic filtering criteria, we find $R=0.515$ like for siblings or parent-offspring’s. We explain this by evolutionary relatedness $R_{ROH>0.1}=0.517$ from ancient inbreeding, $F_{ROH>0.1}=0.258$. This is like $F_{ROH>0.1}$ for Europeans in the 1000GP. We find recent inbreeding $F_{ROH>5}=0.007$ like pedigree inbreeding in the Faroe Islands. Furthermore, we find $F_{ROH>1.5} =0.029$ like for ancient genomes of simple and early complex agriculturalists in West and Central Eurasia, respectively. Similarly, with our advanced filtering criteria, we find $R=0.481$ like $R_{ROH>0.2}=0.485$, the recent inbreeding was $F_{ROH>5}=0.010$ and $F_{ROH>1.5} =0.036$. $F_{ROH>1.5}$ in our study is higher than for the isolated population of endogamous Dalmatians in Croatia and endogamous Orcadians in Orkney. Perhaps the participants in our study were not as unrelated as reported. This is possible, but unlikely given the good records of familial relatedness in the Faroe Islands. With precaution for the small sample, we suggest the population descends from founders of $>99\%$ European and $<1\%$ Admixed American ancestry. The distinct clustering near the central European and British populations of the 1000GP is likely the result of the population isolation and genetic drift. The ancestry is mainly European while the inbreeding is higher compared to European populations and population isolates. The Faroese population has inbreeding more like ancient Europeans. We discovered a bottlenecked and consanguineous population event and estimated it starting in the 1st-4th century as opposed to the oldest archaeological findings from the 4th-6th century. Possibly the founders descended from simple, early complex, or advanced complex agriculturalists, and due to the population isolation, the inbreeding $F_{ROH>1.5}$ remained high. If true, the inbreeding of the modern Faroese population has not decreased as elsewhere, and the population can perhaps be used to study such ancient populations.

## Methods

The samples were sequenced at *x*37 read depth in Cambridge, UK, variants called (Illumina, VN:CASAVA - 1.9.0a1_110909, CASAVA-VariantCalling-2.12a; GRCh37 reference: HumanNCBI_UCSC_XY.fa, HumanNCBI_UCSC_XX.fa) and stored in Variant Call Format (VCFv4.1) files.

On a Biobank server, we used Tabix and BCFtools (both v1.7) [37, 38] to block compress, sort, index, and concatenate the autosome (chr1-chr22) files into one VCF file per sample. We processed the files with BCFtools to get quality-filtered SNPs (TYPE=’snp’, QUAL>30) for our basic filtering criteria, and (TYPE=’snp’, FILTER=’PASS’, QUAL>30, FMT/DPU>10) for our advanced filtering criteria. The FILTER=’PASS’ requires all the CASAVA filters passed (e.g. FILTER ID=QGT20 minimum genotype quality 20, ID=MaxSB strand bias value 10, ID=SitesMaxDept $\approx 90$) and FMT/DPU>10 ensures that the minimum read depth used is 10. We tested several other filters with different minimum and maximum read depth used with little effects on the results (not shown).

With PLINK (v1.90b6.10) [39–41] we converted the files to PLINKs format with missing variant ID’s replaced with unique ID’s [41]. We used a filter (--mind 0.1 --geno 0.1 --hwe 1e-7) for sample-, variant missingness and for Hardy-Weinberg (H-W) equilibrium threshold [41].

We used KING (v2.2.3) for sample quality check (--bysample), which lists the heterozygosity and number of SNPs per sample, and for the relatedness calculations (--kinship) that are robust to population structure and the SNP panel for sample sizes as small as two [31]. We also used KING for quality check by SNP (--bySNP) that at each SNP list the number of homozygoues and heterozygous genotypes and the call rate.

The KING robust pairwise relationship inference assumes HWE across SNPs with the allele frequency *P* within an individual, i.e. $Pr(Aa|P) = 2P(1-P)$. Considering population stratification, *P* may vary between individuals. The genome-wide average heterozygosity ${E}(2P(1-P))$ for individuals *i*, *j* is estimated by

1 $$\begin{aligned} \frac{N^{(i)}_{Aa}}{M_{ij}}, \frac{N^{(j)}_{Aa}}{M_{ij}} \end{aligned}$$

where $N^{(i)}_{Aa}$, $N^{(j)}_{Aa}$ are the total number of heterozygotes for individuals *i*, *j*, respectively, and $M_{ij}$ is the total number of non-missing markers for the pair of individuals because KING only uses markers with genotype data for both individuals for estimating pairwise kinship [31].

Similarly, for two individuals *i*, *j*, the genome-wide average heterozygosity of the pair is estimated by

2 $$\begin{aligned} \frac{N^{(i)}_{Aa} + N^{(j)}_{Aa}}{2M_{ij}} \end{aligned}$$

The $\phi _{ij}$ kinship coefficient is defined as the probability that two random sampled alleles from the two individuals are IBD, and $\pi _{0ij}$, $\pi _{1ij}$ and $\pi _{2ij}$ are the probabilities that the two individuals share zero, one or two alleles IBD, respectively [31]. The kinship coefficient $\phi _{ij}$ is a function of IBD-sharing with relatedness $2\phi _{ij} = \pi _{1ij}/2 + \pi _{2ij}$ [31]. The KING (within-family) pairwise kinship estimator is:

3 $$\begin{aligned} \hat{\phi }_{ij}(t) = \frac{N_{Aa,Aa} - 2N_{AA,aa}}{N^{(i)}_{Aa} + N^{(j)}_{Aa}} \end{aligned}$$

where $N_{Aa,Aa}$, $N_{AA,aa}$ are the total number of SNPs at which both individuals of the pair are heterozygous and different homozygous, respectively [31].

Equation 3 assumes HWE for SNPs with the same underlying allele frequencies while in practice there may be deviations from HWE from e.g., genotyping errors or recent admixture in a mixed population. If the violation of HWE is in the direction of excessive heterozygosity, the robust estimator in Eq. 3 can overestimate the kinship coefficient. To guard against such estimation inflation from departure from individual-level HWE, the smaller of the observed heterozygosity rates $min\left(\frac{N^{(i)}_{Aa}}{M_{ij}}, \frac{N^{(j)}_{Aa}}{M_{ij}}\right)$ is used [31]. Suppose the *i*-th individual has lower heterozygosity than the *j*-th individual. Then the KING (between-family) kinship estimator is:

4 $$\begin{aligned} \hat{\phi }_{ij}(t) = \frac{N_{Aa,Aa} - 2N_{AA,aa}}{2N^{(i)}_{Aa}} + \frac{1}{2} - \frac{1}{4} \frac{N^{(i)}_{Aa} + N^{(j)}_{Aa}}{N^{(i)}_{Aa}} \end{aligned}$$

The estimator in Eqs. 3 and 4 for within- and between-family relationship estimation, respectively, are combined in the KING-robust --kinship method.

We did not explicitly filter or prune the SNPs, because the KING documentation does not recommend to prune, and if sufficient computer memory, neither to filter any rare SNPs that pass quality check [42]. KING relationship inference works well for genome sequence data [42].

The sample VCF files were merged with BCFTools, processed with PLINK similarly to the single samples, and filtered (--geno 0.1 --hwe 1e-7) before further analysis with KING and PLINK.

For *ROH* and *maf* analysis with PLINK, we used the $n=8$ merged file with the eight samples. To study *ROHs* without *maf* filtering or *LD* pruning [34–36] we used the PLINK --homozyg method [36, 40] with default parameters except for the minimum length --homozyg-kb that we varied between 100 and 5000kb. The *maf* results were made with plink --freq [41].

The inbreeding coefficient based on *ROH* is defined by the total length of *ROHs *$\sum {L_{ROH>x}}$ for *ROHs* larger than a minimum length *x* divided by the autosome length $L_{aut}$ covered by the SNPs [20]:

5 $$\begin{aligned} F_{ROH>x} = \frac{\sum {L_{ROH>x}}}{L_{aut}} \end{aligned}$$

The autosome length $L_{aut}=2881033.286$kb was found by adding the GRCh37 reference autosome chromosome lengths from the header info of the BAM files (samtools view -H sorted.realigned.bam).

The minimum *ROH* length *x* may vary depending on the research question. Values of $x= 1, 1.5$Mb are typically used and the $x=1.5$Mb minimum length is preferred to compare population inbreeding of European populations [20, 25, 27], while long minimum lengths of e.g., $x=5$Mb are used to compare with pedigree inbreeding [20]. Sometimes very short values like $x= 0.1$Mb are used [24]. The shorter minimum lengths are used to study inbreeding further back in time when short *ROHs* can be reliably called with high coverage sequency data [20, 24]. To investigate the full spectrum of ancient to recent inbreeding we choose to vary the minimum length between the two extrema of $x=0.1$ and $x=5$Mb in steps of 0.1Mb.

Parental relatedness is two times the inbreeding of an individual assuming their common ancestors are not inbred [43], or equivalently, the coefficient of inbreeding of an individual is the same as the kinship coefficient between the parents of the same individual [44].

Therefore, we infer that given the median or average value of $F_{ROH>x}$ for a sample of individuals, the median or average relatedness of their parents can be estimated by:

6 $$\begin{aligned} R_{ROH>x} = 2F_{ROH>x} \end{aligned}$$

We use $x= 0.1$Mb when comparing $F_{ROH>0.1}$ in our study with inbreeding of the 1000GP populations [24], and for comparing $R_{ROH>0.1}$ with *R* from the KING method based on kinship that cannot discriminate between recent and ancient relatedness. *R* measures pairwise relatedness of the individuals while $R_{ROH>x}$ measures the parental relatedness of the individuals. However, assuming that the average relatedness does not change much in a single generation, the average measures of *R* and $R_{ROH>x}$ are expected to be similar for some value of *x*.

We use $x=1.5$Mb when comparing $F_{ROH>1.5}$ with inbreeding of European populations [20, 25, 27] and $x=5$Mb for comparing $F_{ROH>5}$ with the pedigree inbreeding in the Faroe Islands [23].

We use plots of the number of *ROH* against the total length of *ROH* to search for a bottleneck and consanguineous mating effects [20, 22]. We observe the minimum length $L_{min}$ for which all the sample points have crossed below the diogonal of the plot, and we observe the length $L_{max}$ of *ROHs* for which we find the maximum deviation from the expected diagonal-value in these plots. We assume $L_{min}$ and $L_{max}$ indicate the start and the maximum of the bottleneck and consanguineous mating effects, respectively.

These lengths we use to infer the demographic events in units of generations *g* back in time $t=26.9g$, where we have used the human generation time 26.9 years [45]. We apply the estimation formula for the length of *ROHs* that should follow an exponential distribution with mean $L=100cM/2g$, where *g* is the number of generations since the last common ancestor [46, 47]. For example, if $g=50$ we get $L=1$cM or about 1Mb, and $t=26.9g = 1345$ years back in time. We estimate *g* from the observed *L* of *ROHs*:

7 $$\begin{aligned} \hat{g} = \frac{100cM}{2L} \end{aligned}$$

When calculating the calender year from the years back in time, we count from the year $\sim 1960$ estimated to be the average year of birth of the sampled individuals, instead of using the year of sampling, which was $\sim 2012$. If using the year of sampling, our estimated calender years shift with $\sim {50}$ years up in time.

Finally, population structure inference and ancestry were studied for the $n=8$ merged file with the Euclidean distance Multidimensional Scaling (MDS) method in KING --mds --projection using 2451 1000GP reference samples KGref.bed [48, 49]. The purpose of this methodology was to confirm the ancestry of the samples and to see how they cluster as compared with the reference samples of the 1000GP.

### Supplementary Information

**Additional file 1.**

**Additional file 2.**

**Additional file 3.**

**Additional file 4.**

**Additional file 5.**

**Additional file 6.**

**Additional file 7.**

**Additional file 8.**

**Additional file 9.**

**Additional file 10.**

**Additional file 11.**

## Data Availability

Data collected for the FarGen-infrastructure is available for research up on participants’ re-consent. Researchers will be granted access to de-identified genetic-data and meta-data provided that the project protocol has been approved by the Faroese Scientific Ethical Committee and a template material/data transfer agreement has been signed with the Genetic Biobank of the Faroe Islands in compliance with GDPR. The code to reproduce the results is posted on GitHub: https://github.com/hannesgislason/wg-project.
