# Supplementary material for: SNP heterozygosity, relatedness and inbreeding of whole genomes from the isolated population of the Faroe Islands
Source: BMC Genomics. 2023 Nov 23;24:707. doi: 10.1186/s12864-023-09763-x (PMC10666429; doi:10.1186/s12864-023-09763-x)
Supplement: Supplementary file 1 — Additional file 1. [file 12864_2023_9763_MOESM1_ESM.pdf]

Additional file 1 - Supplementary tables of SNPs, heterozygosity and relatedness

The number of SNPs, the heterozygosity and the relatedness were found from autosome variant files (`.vcf`) containing both SNPs and Indels that we filtered with BCFTools to only include SNPs (`bcftools filter -i TYPE='snp'`). We quality-filtered (`bcftools filter -i QUAL>30`) and merged the sample files (`--bcftools merge`). With PLINK software, we converted the quality-filtered SNPs (`.vcf`) files to PLINK format (`.bed`) with missing variant ID's replaced with unique ID's. We made quality control reports with KING (`king --bysample`, `king --bySNP`) to investigate the missing rate, the number of SNPs and the heterozygosity, and we studied relatedness with KING (`king --kinship`). The effect of merging on the missing rate, N_{SNP}, H and R was studied both before and after applying a PLINK filter for genotype missingness and Hardy-Weinberg (H-W) equilibrium threshold (`--geno 0.1 --hwe 1e-7`). For further details see Methods in the main manuscript.

Table S1.1: Filter (`bcftools filter -i`) used to filter the SNP files before further processing with PLINK and KING.

Autosome files	Filter-text
SNP VCFs	QUAL>30

Table S1.2: The KING quality control report (`--bysample`) for the single samples. Note the low missing rate.

IID	N_SNP	Missing	H
SAMPLE1	3532173	4e-04	0.5983
SAMPLE2	3561096	4e-04	0.6015
SAMPLE3	3554723	4e-04	0.6002
SAMPLE4	3496840	4e-04	0.5737
SAMPLE5	3566139	4e-04	0.5969
SAMPLE6	3557612	4e-04	0.6002
SAMPLE7	3578669	4e-04	0.6043
SAMPLE8	3568947	4e-04	0.6043

Table S1.3: Summary of N_{SNP} on autosomes from the KING quality control (`--bysample`) for the eight single samples ($n = 1$).

n	values	min	q1	med	mean	q3	max	iqr	sd
1	8	3496840	3549086	3559354	3552025	3566841	3578669	17756	26065

Table S1.4: Summary of H on autosomes from the KING quality control (`--bysample`) for the eight single samples ($n = 1$).

n	values	min	q1	med	mean	q3	max	iqr	sd
1	8	0.5737	0.598	0.6002	0.5974	0.6022	0.6043	0.0042	0.0099

Table S1.5: The KING quality control (`--bysample`) for the merged samples. Note the much higher missing rate for the merged samples while both N_{SNP} and H are similar to the values for the single samples. This is because the merging of samples marks the SNPs as missing that are not common to all the samples in the merged dataset.

IID	N_SNP	Missing	H
SAMPLE1	3531455	0.5013	0.5983
SAMPLE2	3559988	0.4973	0.6014
SAMPLE3	3553862	0.4981	0.6001
SAMPLE4	3495666	0.5064	0.5737
SAMPLE5	3564981	0.4966	0.5969
SAMPLE6	3556428	0.4978	0.6002
SAMPLE7	3577394	0.4948	0.6042
SAMPLE8	3567597	0.4962	0.6042

Table S1.6: The KING quality control report (`--bysample`) for the eight merged samples after missing genotype filtering (`--geno 0.1`). Note the large decreases in both N_{SNP} and H as compared to Table S1.5. The missing column is 0 since all missing genotypes have been filtered out and N_{SNP} becomes equal for all the samples.

IID	N_SNP	Missing	H
SAMPLE1	1136546	0	0.2861
SAMPLE2	1136546	0	0.2992
SAMPLE3	1136546	0	0.2836
SAMPLE4	1136546	0	0.2812
SAMPLE5	1136546	0	0.2928
SAMPLE6	1136546	0	0.2920
SAMPLE7	1136546	0	0.2968
SAMPLE8	1136546	0	0.2960

Table S1.7: Summary of N_{SNP} on autosomes from the KING quality control (`--bysample`) for the merged samples ($n = 8$) before (first row) and after (second row) missing genotypes filtering. Note the similarity of N_{SNP} before the filtering with N_{SNP} for single samples in Table S1.3.

n	values	min	q1	med	mean	q3	max	iqr	sd
8	8	3495666	3548260	3558208	3550921	3565635	3577394	17375	25994
8	8	1136546	1136546	1136546	1136546	1136546	1136546	0	0

Table S1.8: Summary of the heterozygosity H on autosomes from the KING quality control (`--bysample`) for the merged samples ($n = 8$) before and after missing genotypes filtering. Note the similarity of H before the filtering with H for single samples in Table S1.4

n	values	min	q1	med	mean	q3	max	iqr	sd
8	8	0.5737	0.5980	0.6002	0.5974	0.6021	0.6042	0.0042	0.0099
8	8	0.2812	0.2855	0.2924	0.2910	0.2962	0.2992	0.0107	0.0066

Table S1.9: Summary of the pairwise relatedness R on autosomes from the KING (`--kinship`) report for the eight merged samples ($n = 8$) before and after missing genotypes filtering. Here, the number of values is 28 since there are 28 possible pairwise relationships between eight samples.

n	values	min	q1	med	mean	q3	max	iqr	sd
8	28	0.6442	0.6628	0.6694	0.6714	0.6719	0.8222	0.0091	0.0310
8	28	0.5016	0.5102	0.5148	0.5239	0.5258	0.7312	0.0156	0.0415

Table S1.10: Summary of the N_{SNP} from the KING (`--kinship`) report used to calculate pairwise relatedness for the eight merged samples before and after missing genotypes filtering. Here, N_{SNP} is the number of SNPs shared between the two samples of each relationship. It is lower than the N_{SNP} of both the single samples (Table S1.3) and the merged samples (Table S1.7) before missing genotype filtering. It is the same as the N_{SNP} for merged samples after missing genotype filtering where all the samples share the same SNPs.

n	values	min	q1	med	mean	q3	max	iqr	sd
8	28	2310639	2336570	2348854	2365699	2359269	2885319	22699	103284
8	28	1136546	1136546	1136546	1136546	1136546	1136546	0	0

Table S1.11: Summary of the KING quality report (`--bySNP`) for the eight merged samples before missing genotype filtering. The summary is grouped by the number of samples N with non-missing genotypes. The H across SNPs is highest for the SNPs only called in one sample and lowest for the SNPs called in all samples.

N	CallRate	SNPs	Genotypes	Aa	H
1	0.125	1724918	1724918	1595874	0.9252
2	0.250	1031330	2062660	1841243	0.8927
3	0.375	770323	2310969	1966315	0.8509
4	0.500	654173	2616692	2103921	0.8040
5	0.625	587430	2937150	2180339	0.7423
6	0.750	571066	3426396	2271963	0.6631
7	0.875	605174	4236218	2366225	0.5586
8	1.000	1136546	9092368	2645501	0.2910

Table S1.12: Summary of the KING quality report (`--bySNP`) for the merged samples both before and after missing genotype filtering (`--geno 0.1`). The overall H is calculated from the number of heterozygous genotypes divided by the total number of genotypes. Without the missing genotype filter it is equal to the mean H for the single samples (Table S1.4). With the filter it is the same as H for the SNPs called in all samples (Table S1.11, last row).

N	SNPs	Genotypes	Aa	H
1-8	7080960	28407371	16971381	0.5974
8	1136546	9092368	2645501	0.2910
