# Supplementary material for: SNP heterozygosity, relatedness and inbreeding of whole genomes from the isolated population of the Faroe Islands
Source: BMC Genomics. 2023 Nov 23;24:707. doi: 10.1186/s12864-023-09763-x (PMC10666429; doi:10.1186/s12864-023-09763-x)
Supplement: Supplementary file 3 — Additional file 3. [file 12864_2023_9763_MOESM3_ESM.pdf]

Additional file 3 - Supplementary table and plot of the minor allele frequency distribution

Table S3.1: Filter (`bcftools filter -i`) used to filter the SNP files before further processing with PLINK.

Autosome files	Filter-text
SNP VCFs	QUAL>30

Table S3.2: The maf-distribution of 1136546 SNPs for the eight merged samples from the Faroe Islands made with PLINK (`plink --freq`), and summarised with counts and proportions for each minor allele frequency. With eight samples and 16 alleles at each SNP position there are nine possible minor allele frequencies: $(0-8)/16 = 0.0000, 0.0625, 0.1250, 0.1875, 0.2500, 0.3125, 0.3750, 0.4375, 0.5000$.

maf	count	proportion
0.0000	379415	0.3338
0.0625	146428	0.1288
0.1250	145813	0.1283
0.1875	135406	0.1191
0.2500	111420	0.0980
0.3125	84796	0.0746
0.3750	53253	0.0469
0.4375	28069	0.0247
0.5000	51946	0.0457

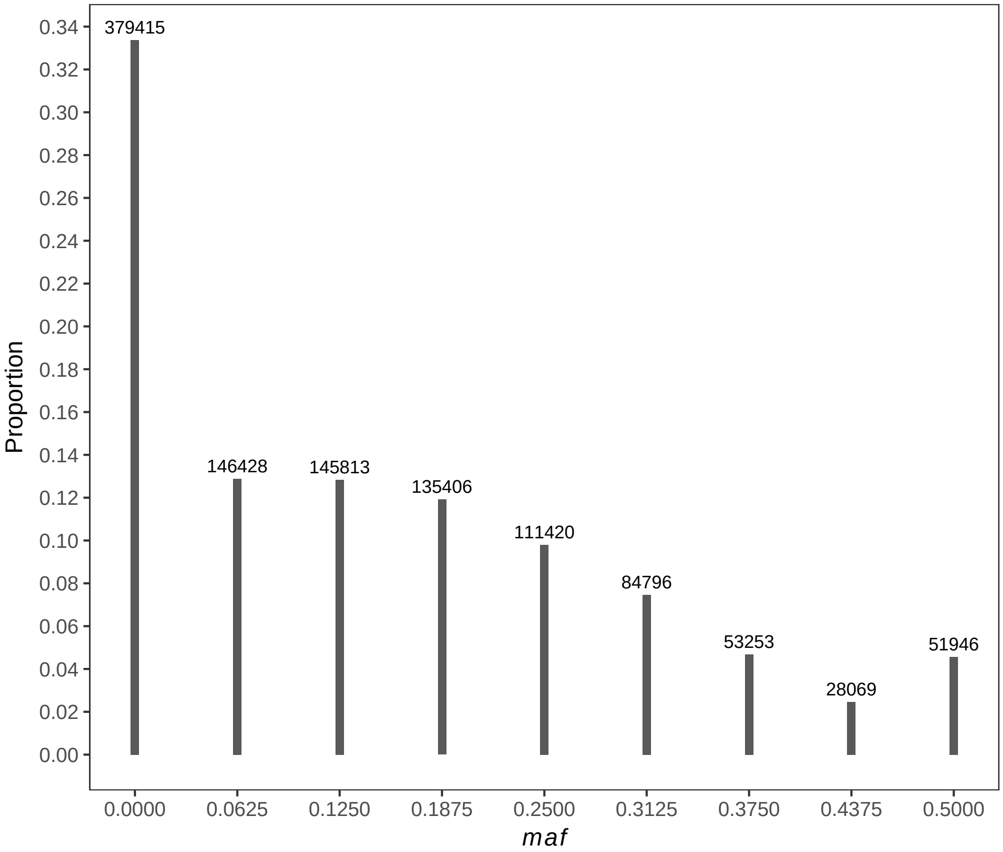

Figure S3.1: The maf-distribution of 1136546 SNPs for the eight merged samples from the Faroe Islands made with PLINK and summarised with counts and proportions for each minor allele frequency. The proportions are on the y-scale and the counts above the bars.
