# Supplementary material for: SNP heterozygosity, relatedness and inbreeding of whole genomes from the isolated population of the Faroe Islands
Source: BMC Genomics. 2023 Nov 23;24:707. doi: 10.1186/s12864-023-09763-x (PMC10666429; doi:10.1186/s12864-023-09763-x)
Supplement: Supplementary file 4 — Additional file 4. [file 12864_2023_9763_MOESM4_ESM.pdf]

Additional file 4 - Supplementary ancestry analysis

Table S4.1: Filter (`bcftools filter -i`) used to filter the SNP files before further processing with KING.

Autosome files	Filter-text
SNP VCFs	QUAL>30

Table S4.2: Summary of the KING (`--mds --projection`) inferred ancestry using 700832 autosome SNPs of the eight samples from the Faroe Islands projected together with a 1000GP reference (KGref.bed, 2451 samples).

Ancestry	values	parameter	min	q1	med	mean	q3	max	iqr	sd
EUR	8	Pr_Anc	0.994	0.9968	0.997	0.9969	0.9972	0.999	0.0005	0.0015
AMR	8	Pr_2nd	0.001	0.0010	0.001	0.0018	0.0023	0.004	0.0013	0.0012

Table S4.3: Summary of the KING (`--mds --projection`) 20 MDS PCs for the eight samples from the Faroe Islands. The first 20 PCs are able to explain 19.3% of total variance. The proportion of total variance explained (%) by each PC is: 11.3 4.4 1.1 0.8 0.2 0.1 0.1 0.1 0.1 0.1 0.1 0.1 0.1 0.1 0.1 0.1 0.1 0.1 0.1 0.1.

PC	values	min	q1	med	mean	q3	max	iqr	sd
PC1	8	-0.0167	-0.0166	-0.0162	-0.0162	-0.0160	-0.0154	0.0006	0.0005
PC2	8	-0.0297	-0.0292	-0.0290	-0.0290	-0.0286	-0.0283	0.0005	0.0005
PC3	8	0.0149	0.0151	0.0160	0.0161	0.0166	0.0187	0.0015	0.0013
PC4	8	0.0163	0.0175	0.0186	0.0186	0.0192	0.0216	0.0017	0.0017
PC5	8	-0.0079	-0.0057	-0.0051	-0.0046	-0.0027	-0.0020	0.0030	0.0021
PC6	8	0.0140	0.0147	0.0171	0.0178	0.0185	0.0264	0.0038	0.0041
PC7	8	-0.0090	-0.0069	-0.0048	-0.0042	-0.0016	0.0015	0.0053	0.0039
PC8	8	-0.0105	-0.0072	-0.0054	-0.0036	-0.0020	0.0104	0.0052	0.0065
PC9	8	-0.0076	-0.0059	-0.0040	-0.0033	-0.0004	0.0018	0.0055	0.0036
PC10	8	-0.0066	-0.0043	-0.0025	0.0024	0.0123	0.0156	0.0166	0.0094
PC11	8	-0.0151	-0.0036	0.0043	0.0016	0.0088	0.0100	0.0124	0.0088
PC12	8	-0.0071	-0.0030	0.0009	0.0020	0.0085	0.0100	0.0115	0.0065
PC13	8	-0.0087	-0.0015	-0.0006	0.0008	0.0022	0.0102	0.0036	0.0062
PC14	8	-0.0068	0.0034	0.0068	0.0055	0.0097	0.0113	0.0064	0.0060
PC15	8	-0.0106	-0.0053	-0.0006	0.0003	0.0037	0.0189	0.0090	0.0093
PC16	8	-0.0116	-0.0066	-0.0050	-0.0038	0.0012	0.0024	0.0077	0.0049
PC17	8	-0.0129	-0.0014	0.0058	0.0030	0.0086	0.0116	0.0100	0.0081
PC18	8	-0.0079	0.0011	0.0046	0.0053	0.0084	0.0220	0.0073	0.0088
PC19	8	-0.0087	0.0004	0.0034	0.0034	0.0075	0.0125	0.0071	0.0065
PC20	8	-0.0220	-0.0141	-0.0100	-0.0102	-0.0088	0.0097	0.0052	0.0099

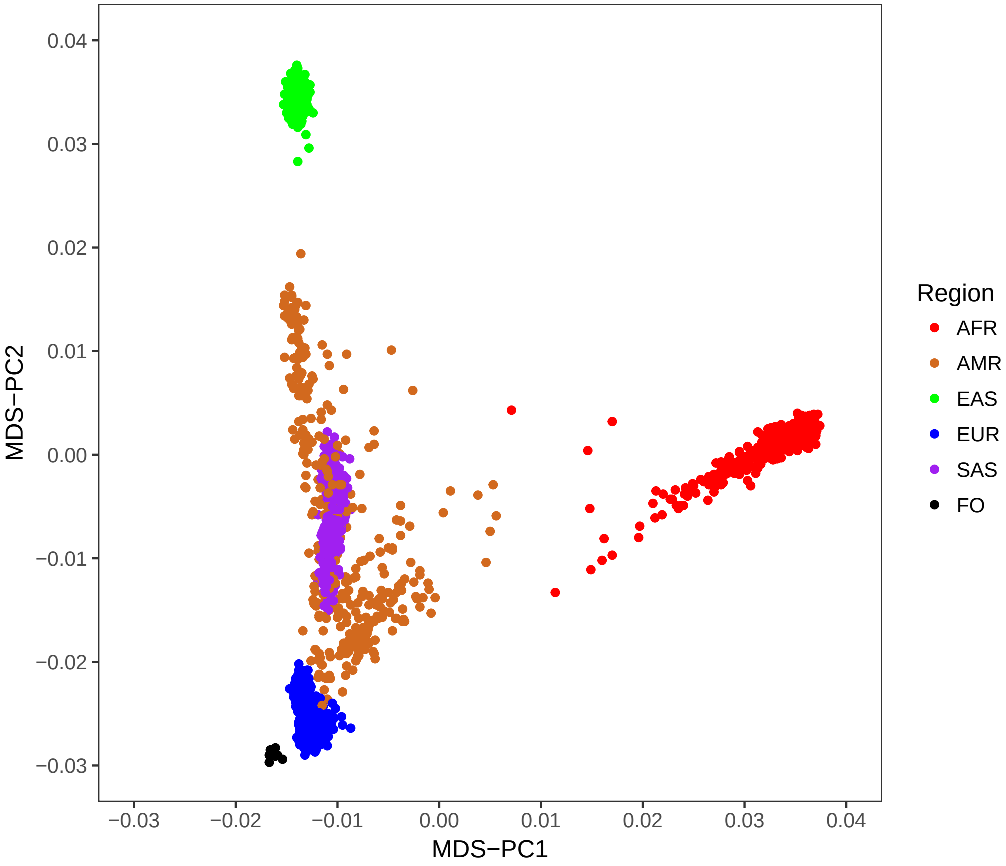

Figure S4.1: The multidimensional scaling (MDS) principal components (PC) of the samples from the Faroe Islands projected to the PC space of the 1000GP. The samples from FO (Faroe Islands, 8 samples) were projected together with a 1000GP reference (KGref.bed, 2451 samples): African (AFR, 637 samples), Admixed American (AMR, 336), East Asian (EAS, 498), European (EUR, 500) and South Asian (SAS, 480).

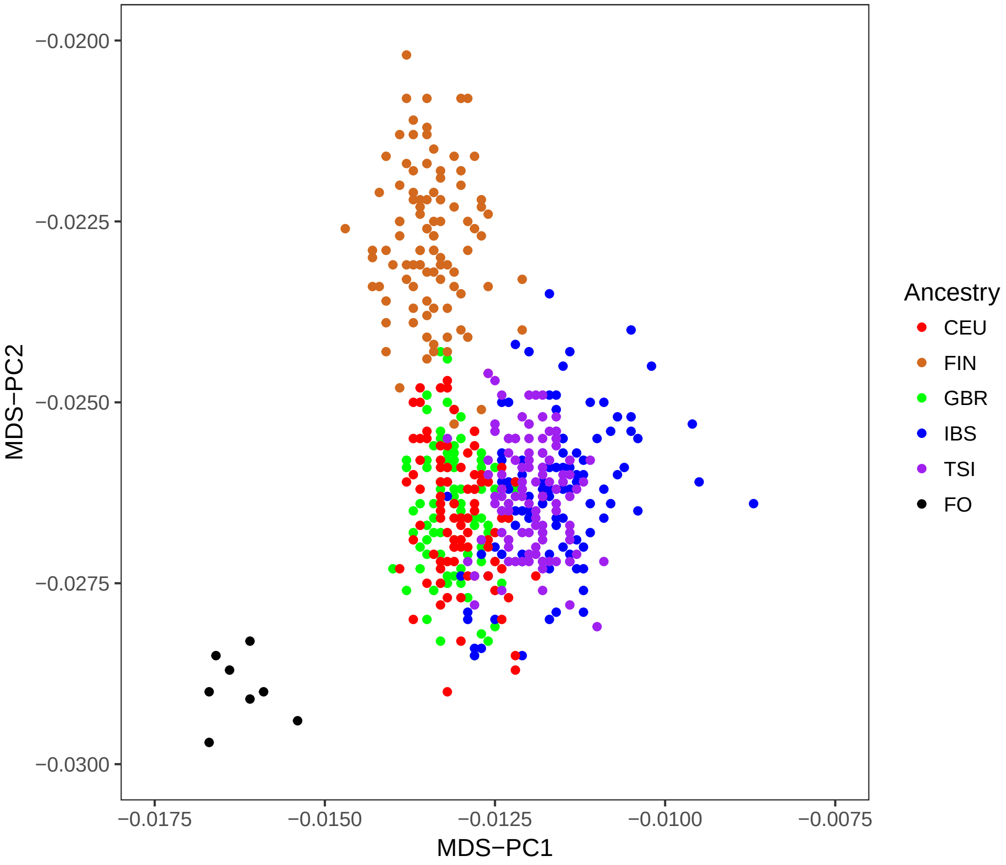

Figure S4.2: The multidimensional scaling (MDS) principal components (PC) of the samples from the Faroe Islands projected to the PC space of samples with European ancestry from the 1000GP. The FO (Faroe Islands, 8 samples) were projected together with populations of European ancestry: CEU (Utah residents with Northen and Western European ancestry, 98 samples), FIN (Finish in Finland, 99 samples), GBR (British in England and Scotland, 89 samples), IBS (Iberian population in Spain, 107 samples), TSI (Toscani in Italy, 107 samples).
