# Supplementary material for: SNP heterozygosity, relatedness and inbreeding of whole genomes from the isolated population of the Faroe Islands
Source: BMC Genomics. 2023 Nov 23;24:707. doi: 10.1186/s12864-023-09763-x (PMC10666429; doi:10.1186/s12864-023-09763-x)
Supplement: Supplementary file 5 — Additional file 5. [file 12864_2023_9763_MOESM5_ESM.pdf]

Additional file 5 - Supplementary tables of SNPs, heterozygosity and relatedness

The number of SNPs, the heterozygosity and the relatedness were found from autosome variant files (`.vcf`) containing both SNPs and Indels that we filtered with BCFTools to only include SNPs (`bcftools filter -i TYPE='snp'`). We quality-filtered (`bcftools filter -i FILTER='PASS' & QUAL>30 & FMT/DPU>10`) and merged the sample files (`--bcftools merge`). With PLINK software, we converted the quality-filtered SNPs (`.vcf`) files to PLINK format (`.bed`) with missing variant ID's replaced with unique ID's. We made quality control reports with KING (`king --bysample`, `king --bySNP`) to investigate the missing rate, the number of SNPs and the heterozygosity, and we studied relatedness with KING (`king --kinship`). The effect of merging on the missing rate, N_{SNP}, H and R was studied both before and after applying a PLINK filter for genotype missingness and Hardy-Weinberg (H-W) equilibrium threshold (`--geno 0.1 --hwe 1e-7`). For further details see Methods in the main manuscript.

Table S5.1: Filter (`bcftools filter -i`) used to filter the SNP files before further processing with PLINK and KING.

Autosome files	Filter-text
SNP VCFs	FILTER='PASS' & QUAL>30 & FMT/DPU>10

Table S5.2: The KING quality control report (`--bysample`) for the single samples. Note the low missing rate.

IID	N_SNP	Missing	H
SAMPLE1	3210983	3e-04	0.6063
SAMPLE2	3244066	3e-04	0.6091
SAMPLE3	3241718	3e-04	0.6071
SAMPLE4	3172132	3e-04	0.5809
SAMPLE5	3249372	3e-04	0.6040
SAMPLE6	3242912	3e-04	0.6078
SAMPLE7	3264288	3e-04	0.6119
SAMPLE8	3250979	3e-04	0.6113

Table S5.3: Summary of N_{SNP} on autosomes from the KING quality control (`--bysample`) for the eight single samples ($n = 1$).

n	values	min	q1	med	mean	q3	max	iqr	sd
1	8	3172132	3234034	3243489	3234556	3249774	3264288	15740	29368

Table S5.4: Summary of H on autosomes from the KING quality control (`--bysample`) for the eight single samples ($n = 1$).

n	values	min	q1	med	mean	q3	max	iqr	sd
1	8	0.5809	0.6057	0.6074	0.6048	0.6096	0.6119	0.0039	0.01

Table S5.5: The KING quality control (`--bysample`) for the merged samples. Note the much higher missing rate for the merged samples while both N_{SNP} and H are similar to the values for the single samples. This is because the merging of samples marks the SNPs as missing that are not common to all the samples in the merged dataset.

IID	N_SNP	Missing	H
SAMPLE1	3210469	0.5038	0.6063
SAMPLE2	3243247	0.4987	0.6090
SAMPLE3	3241119	0.4990	0.6071
SAMPLE4	3171262	0.5098	0.5809
SAMPLE5	3248529	0.4979	0.6040
SAMPLE6	3241995	0.4989	0.6077
SAMPLE7	3263351	0.4956	0.6118
SAMPLE8	3249991	0.4977	0.6113

Table S5.6: The KING quality control report (`--bysample`) for the eight merged samples after missing genotype filtering (`--geno 0.1`). Note the large decreases in both N_{SNP} and H as compared to Table S5.5. The missing column is 0 since all missing genotypes have been filtered out and N_{SNP} becomes equal for all the samples.

IID	N_SNP	Missing	H
SAMPLE1	1002681	0	0.2757
SAMPLE2	1002681	0	0.2888
SAMPLE3	1002681	0	0.2736
SAMPLE4	1002681	0	0.2705
SAMPLE5	1002681	0	0.2823
SAMPLE6	1002681	0	0.2818
SAMPLE7	1002681	0	0.2873
SAMPLE8	1002681	0	0.2859

Table S5.7: Summary of N_{SNP} on autosomes from the KING quality control (`--bysample`) for the merged samples ($n = 8$) before (first row) and after (second row) missing genotypes filtering. Note the similarity of N_{SNP} before the filtering with N_{SNP} for single samples in Table S5.3.

n	values	min	q1	med	mean	q3	max	iqr	sd
8	8	3171262	3233456	3242621	3233745	3248894	3263351	15438	29320
8	8	1002681	1002681	1002681	1002681	1002681	1002681	0	0

Table S5.8: Summary of the heterozygosity H on autosomes from the KING quality control (`--bysample`) for the merged samples ($n = 8$) before and after missing genotypes filtering. Note the similarity of H before the filtering with H for single samples in Table S5.4

n	values	min	q1	med	mean	q3	max	iqr	sd
8	8	0.5809	0.6057	0.6074	0.6048	0.6096	0.6118	0.0039	0.0100
8	8	0.2705	0.2752	0.2821	0.2807	0.2862	0.2888	0.0111	0.0068

Table S5.9: Summary of the pairwise relatedness R on autosomes from the KING (`--kinship`) report for the eight merged samples ($n = 8$) before and after missing genotypes filtering. Here, the number of values is 28 since there are 28 possible pairwise relationships between eight samples.

n	values	min	q1	med	mean	q3	max	iqr	sd
8	28	0.6398	0.6598	0.6667	0.6682	0.6688	0.8238	0.0090	0.0320
8	28	0.4662	0.4762	0.4807	0.4911	0.4931	0.7166	0.0169	0.0452

Table S5.10: Summary of the N_{SNP} from the KING (`--kinship`) report used to calculate pairwise relatedness for the eight merged samples before and after missing genotypes filtering. Here, N_{SNP} is the number of SNPs shared between the two samples of each relationship. It is lower than the N_{SNP} of both the single samples (Table S5.3) and the merged samples (Table S5.7) before missing genotype filtering. It is the same as the N_{SNP} for merged samples after missing genotype filtering where all the samples share the same SNPs.

n	values	min	q1	med	mean	q3	max	iqr	sd
8	28	2088712	2112618	2128413	2141734	2138904	2618966	26286	95364
8	28	1002681	1002681	1002681	1002681	1002681	1002681	0	0

Table S5.11: Summary of the KING quality report (`--bySNP`) for the eight merged samples before missing genotype filtering. The summary is grouped by the number of samples N with non-missing genotypes. The H across SNPs is highest for the SNPs only called in one sample and lowest for the SNPs called in all samples.

N	CallRate	SNPs	Genotypes	Aa	H
1	0.125	1566593	1566593	1492943	0.9530
2	0.250	948901	1897802	1730661	0.9119
3	0.375	710234	2130702	1844802	0.8658
4	0.500	605906	2423624	1974821	0.8148
5	0.625	544164	2720820	2041908	0.7505
6	0.750	530035	3180210	2120068	0.6666
7	0.875	561252	3928764	2189581	0.5573
8	1.000	1002681	8021448	2251935	0.2807

Table S5.12: Summary of the KING quality report (`--bySNP`) for the merged samples both before and after missing genotype filtering (`--geno 0.1`). The overall H is calculated from the number of heterozygous genotypes divided by the total number of genotypes. Without the missing genotype filter it is equal to the mean H for the single samples (Table S5.4). With the filter it is the same as H for the SNPs called in all samples (Table S5.11, last row).

N	SNPs	Genotypes	Aa	H
1-8	6469766	25869963	15646719	0.6048
8	1002681	8021448	2251935	0.2807
