# Supplementary material for: SNP heterozygosity, relatedness and inbreeding of whole genomes from the isolated population of the Faroe Islands
Source: BMC Genomics. 2023 Nov 23;24:707. doi: 10.1186/s12864-023-09763-x (PMC10666429; doi:10.1186/s12864-023-09763-x)
Supplement: Supplementary file 7 — Additional file 7. [file 12864_2023_9763_MOESM7_ESM.pdf]

Additional file 7 - Supplementary table and plot of the minor allele frequency distribution

Table S7.1: Filter (`bcftools filter -i`) used to filter the SNP files before further processing with PLINK.

Autosome files	Filter-text
SNP VCFs	FILTER='PASS' & QUAL>30 & FMT/DPU>10

Table S7.2: The maf-distribution of 1002681 SNPs for the eight merged samples from the Faroe Islands made with PLINK (`plink --freq`), and summarised with counts and proportions for each minor allele frequency. With eight samples and 16 alleles at each SNP position there are nine possible minor allele frequencies: $(0-8)/16 = 0.0000, 0.0625, 0.1250, 0.1875, 0.2500, 0.3125, 0.3750, 0.4375, 0.5000$.

maf	count	proportion
0.0000	335206	0.3343
0.0625	130633	0.1303
0.1250	131960	0.1316
0.1875	123735	0.1234
0.2500	102283	0.1020
0.3125	77649	0.0774
0.3750	48255	0.0481
0.4375	24410	0.0243
0.5000	28550	0.0285

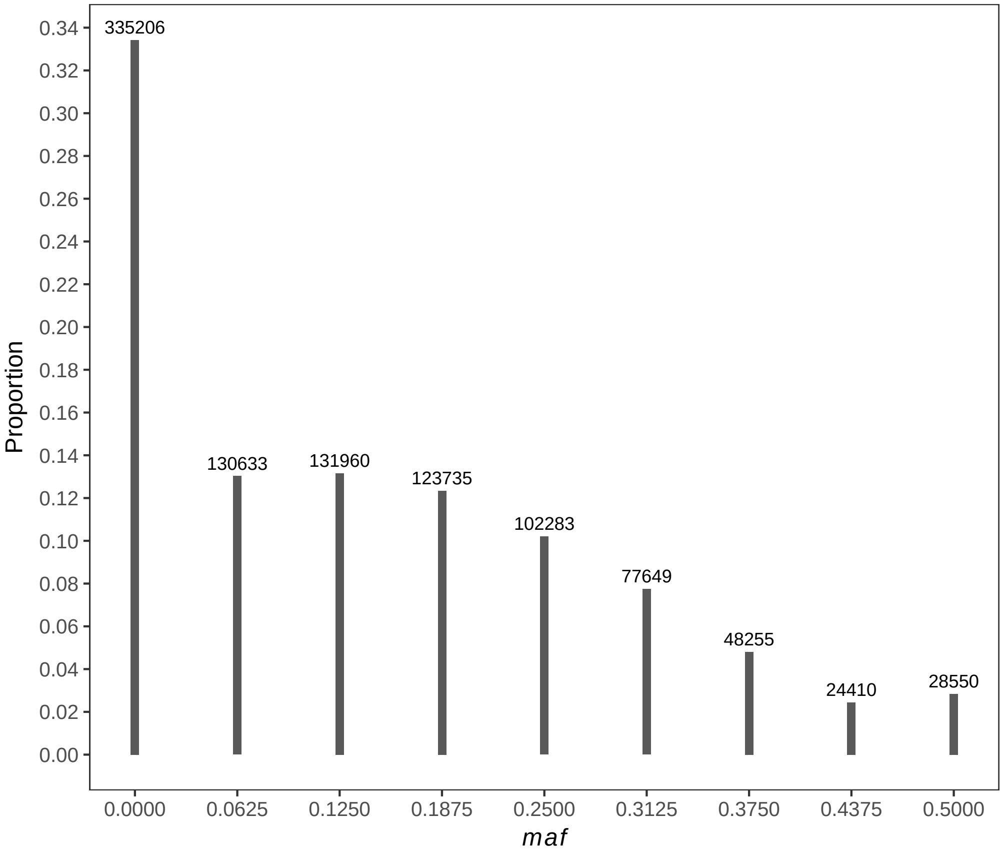

Figure S7.1: The maf-distribution of 1002681 SNPs for the eight merged samples from the Faroe Islands made with PLINK and summarised with counts and proportions for each minor allele frequency. The proportions are on the y-scale and the counts above the bars.
