# Supplementary material for: SNP heterozygosity, relatedness and inbreeding of whole genomes from the isolated population of the Faroe Islands
Source: BMC Genomics. 2023 Nov 23;24:707. doi: 10.1186/s12864-023-09763-x (PMC10666429; doi:10.1186/s12864-023-09763-x)
Supplement: Supplementary file 8 — Additional file 8. [file 12864_2023_9763_MOESM8_ESM.pdf]

Additional file 8 - Supplementary ancestry analysis

Table S8.1: Filter (`bcftools filter -i`) used to filter the SNP files before further processing with KING.

Autosome files	Filter-text
SNP VCFs	FILTER='PASS' & QUAL>30 & FMT/DPU>10

Table S8.2: Summary of the KING (`--mds --projection`) inferred ancestry using 647899 autosome SNPs of the eight samples from the Faroe Islands projected together with a 1000GP reference (KGref.bed, 2451 samples).

Ancestry	values	parameter	min	q1	med	mean	q3	max	iqr	sd
EUR	8	Pr__Anc	0.995	0.9968	0.9975	0.9972	0.9980	0.999	0.0012	0.0013
AMR	8	Pr__2nd	0.001	0.0010	0.0015	0.0018	0.0023	0.003	0.0013	0.0009

Table S8.3: Summary of the KING (`--mds --projection`) 20 MDS PCs for the eight samples from the Faroe Islands. The first 20 PCs are able to explain 19.3% of total variance. The proportion of total variance explained (%) by each PC is: 11.3 4.4 1.1 0.8 0.2 0.1 0.1 0.1 0.1 0.1 0.1 0.1 0.1 0.1 0.1 0.1 0.1 0.1 0.1 0.1.

PC	values	min	q1	med	mean	q3	max	iqr	sd
PC1	8	-0.0167	-0.0166	-0.0162	-0.0162	-0.0160	-0.0153	0.0006	0.0005
PC2	8	-0.0297	-0.0293	-0.0290	-0.0290	-0.0287	-0.0283	0.0005	0.0005
PC3	8	0.0149	0.0150	0.0160	0.0161	0.0165	0.0186	0.0014	0.0012
PC4	8	0.0164	0.0176	0.0184	0.0186	0.0192	0.0216	0.0016	0.0016
PC5	8	-0.0076	-0.0056	-0.0053	-0.0046	-0.0026	-0.0019	0.0029	0.0020
PC6	8	0.0141	0.0151	0.0171	0.0178	0.0184	0.0267	0.0033	0.0041
PC7	8	-0.0073	-0.0048	-0.0032	-0.0029	-0.0017	0.0042	0.0032	0.0036
PC8	8	-0.0097	0.0008	0.0062	0.0033	0.0074	0.0082	0.0066	0.0061
PC9	8	-0.0083	-0.0065	-0.0043	-0.0034	-0.0001	0.0028	0.0064	0.0042
PC10	8	-0.0170	-0.0126	0.0022	-0.0031	0.0041	0.0047	0.0167	0.0093
PC11	8	-0.0161	-0.0049	0.0040	0.0012	0.0081	0.0117	0.0130	0.0094
PC12	8	-0.0055	-0.0036	0.0007	0.0020	0.0079	0.0105	0.0115	0.0064
PC13	8	-0.0080	-0.0005	0.0000	0.0012	0.0028	0.0109	0.0034	0.0055
PC14	8	-0.0064	0.0013	0.0054	0.0047	0.0087	0.0120	0.0074	0.0063
PC15	8	-0.0072	-0.0060	-0.0016	0.0012	0.0060	0.0200	0.0120	0.0094
PC16	8	-0.0071	-0.0016	0.0016	0.0004	0.0033	0.0067	0.0048	0.0048
PC17	8	-0.0097	-0.0059	0.0002	0.0014	0.0088	0.0140	0.0148	0.0095
PC18	8	-0.0114	0.0036	0.0054	0.0061	0.0106	0.0221	0.0070	0.0095
PC19	8	-0.0048	-0.0028	-0.0002	0.0002	0.0015	0.0100	0.0043	0.0047
PC20	8	-0.0167	-0.0119	-0.0094	-0.0058	-0.0008	0.0154	0.0111	0.0105

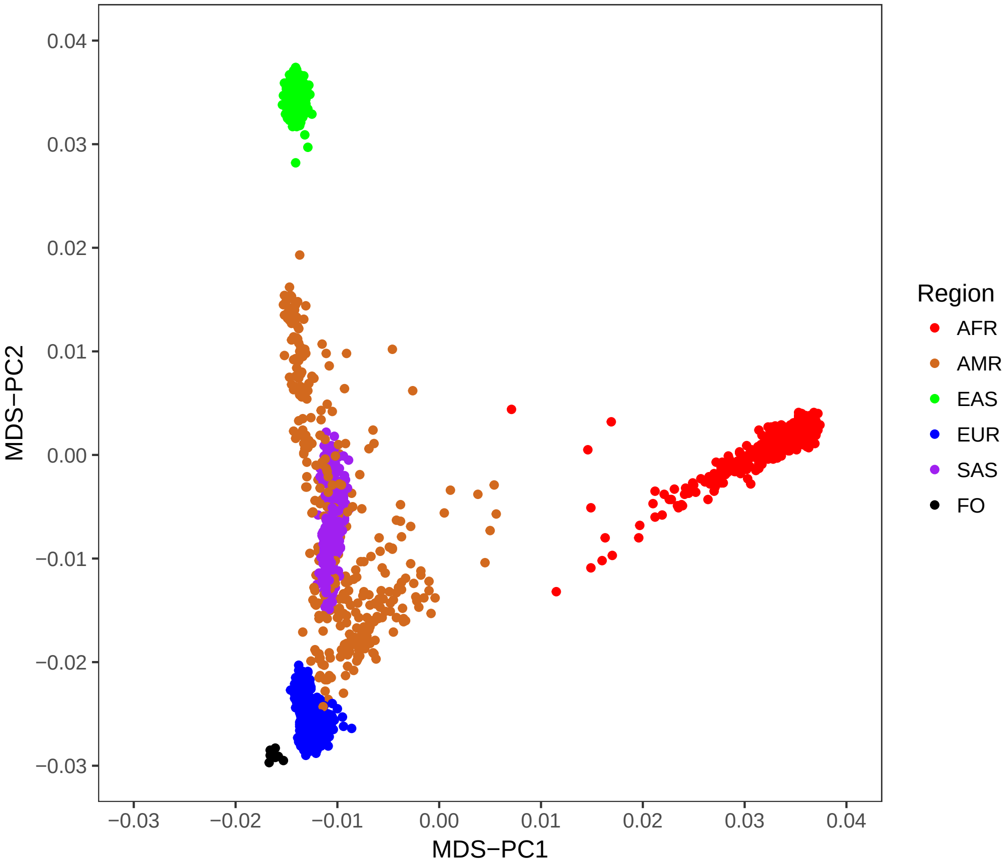

Figure S8.1: The multidimensional scaling (MDS) principal components (PC) of the samples from the Faroe Islands projected to the PC space of the 1000GP. The samples from FO (Faroe Islands, 8 samples) were projected together with a 1000GP reference (KGref.bed, 2451 samples): African (AFR, 637 samples), Admixed American (AMR, 336), East Asian (EAS, 498), European (EUR, 500) and South Asian (SAS, 480).

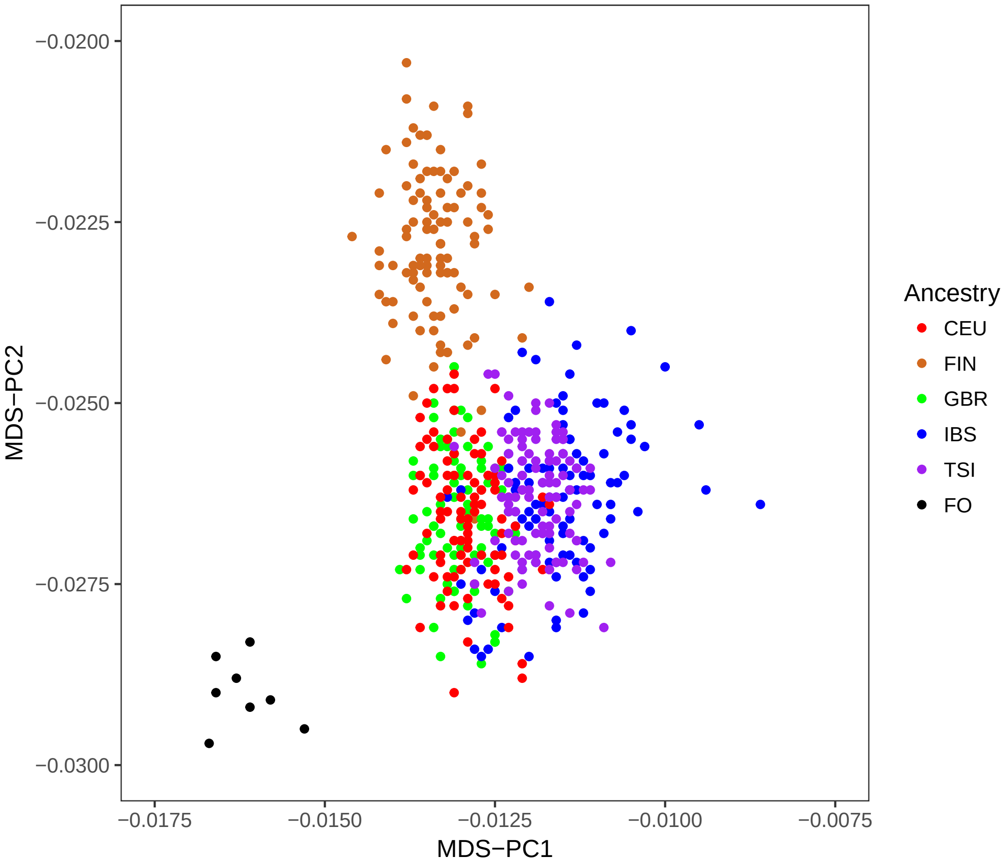

Figure S8.2: The multidimensional scaling (MDS) principal components (PC) of the samples from the Faroe Islands projected to the PC space of samples with European ancestry from the 1000GP. The FO (Faroe Islands, 8 samples) were projected together with populations of European ancestry: CEU (Utah residents with Northen and Western European ancestry, 98 samples), FIN (Finish in Finland, 99 samples), GBR (British in England and Scotland, 89 samples), IBS (Iberian population in Spain, 107 samples), TSI (Toscani in Italy, 107 samples).
