# Supplementary material for: SNP heterozygosity, relatedness and inbreeding of whole genomes from the isolated population of the Faroe Islands
Source: BMC Genomics. 2023 Nov 23;24:707. doi: 10.1186/s12864-023-09763-x (PMC10666429; doi:10.1186/s12864-023-09763-x)
Supplement: Supplementary file 10 — Additional file 10. [file 12864_2023_9763_MOESM10_ESM.pdf]

Additional file 10 - Supplementary tables and figures of *ROH* with McQuillan2008 *ROH* parameters: –homozyg –homozyg-snp 25 –homozyg-kb hkb –homozyg-gap 100 for minimum *ROH* > 500kb.

Table S10.1: Filter (`bcftools filter -i`) used to filter the SNP files before further processing with PLINK.

Autosome files	Filter-text
SNP VCFs	FILTER='PASS' & QUAL>30 & FMT/DPU>10

Table S10.2: Summary of the number of runs of homozygosity *NSEG* on autosomes from the PLINK report (`--homozyg --homozyg-snp 25 --homozyg-kb hkb --homozyg-gap 100`) for variable minimum *ROH* length (Mb).

Mb	distinct	min	q1	med	mean	q3	max	iqr	sd
0.5	7	449	456.0	478.0	484.0	503.5	531	47.5	32.5
0.6	8	286	303.5	319.0	321.0	340.5	354	37.0	23.6
0.7	8	205	208.2	214.0	221.4	234.5	253	26.2	17.6
0.8	8	135	145.5	151.5	157.5	168.0	190	22.5	19.1
0.9	8	102	105.8	110.0	117.2	121.8	152	16.0	17.7
1.0	7	69	77.2	84.0	87.5	94.8	119	17.5	17.0
1.1	8	51	53.5	58.5	65.0	68.2	103	14.8	17.7
1.2	7	37	40.0	45.0	51.5	55.0	89	15.0	17.5
1.3	8	27	32.5	38.5	41.9	44.0	77	11.5	16.0
1.4	8	21	24.2	27.0	33.5	37.8	67	13.5	15.4
1.5	7	14	18.8	23.0	27.6	31.5	62	12.8	15.4
1.6	8	12	16.8	19.5	24.1	24.8	60	8.0	15.3
1.7	8	8	14.0	16.5	21.0	21.5	55	7.5	14.8
1.8	7	8	11.0	13.5	17.6	17.2	49	6.2	13.4
1.9	7	7	9.5	12.5	15.9	14.5	45	5.0	12.3
2.0	8	5	7.8	10.0	13.4	13.0	39	5.2	10.9
2.1	7	5	6.0	9.5	12.1	11.8	36	5.8	10.1
2.2	8	4	5.8	8.5	11.1	11.2	34	5.5	9.7
2.3	7	3	4.8	7.5	9.5	8.8	30	4.0	8.7
2.4	7	2	4.8	7.0	9.1	8.5	30	3.8	8.8
2.5	6	1	3.8	7.0	8.4	7.8	28	4.0	8.4
2.6	7	1	3.5	6.0	7.4	7.2	25	3.8	7.5
2.7	7	1	2.5	5.5	6.8	7.2	23	4.8	7.1
2.8	6	1	2.5	4.5	6.0	5.5	22	3.0	6.8
2.9	6	1	2.5	4.0	5.6	5.2	21	2.8	6.5
3.0	6	1	2.5	3.5	5.1	5.2	18	2.8	5.5
3.1	6	0	2.5	3.5	4.5	4.2	16	1.8	4.9
3.2	5	0	2.2	3.0	4.0	4.2	14	2.0	4.4
3.3	5	0	1.5	2.0	3.1	3.2	12	1.8	3.8
3.4	5	0	1.5	2.0	3.1	3.2	12	1.8	3.8

Mb	distinct	min	q1	med	mean	q3	max	iqr	sd
3.5	5	0	1.5	2.0	3.1	3.2	12	1.8	3.8
3.6	5	0	1.5	2.0	3.0	3.2	11	1.8	3.5
3.7	5	0	1.5	2.0	2.9	3.2	10	1.8	3.2
3.8	6	0	0.8	2.0	2.5	3.2	8	2.5	2.6
3.9	6	0	0.8	2.0	2.2	3.2	6	2.5	2.1
4.0	5	0	0.0	2.0	2.1	3.2	6	3.2	2.2
4.1	5	0	0.0	2.0	2.1	3.2	6	3.2	2.2
4.2	3	0	0.0	2.0	1.8	2.5	4	2.5	1.7
4.3	4	0	0.0	1.5	1.6	2.5	4	2.5	1.7
4.4	5	0	0.0	1.5	1.5	2.2	4	2.2	1.5
4.5	5	0	0.0	1.5	1.5	2.2	4	2.2	1.5
4.6	5	0	0.0	1.0	1.4	2.2	4	2.2	1.5
4.7	4	0	0.0	1.0	1.2	1.5	4	1.5	1.5
4.8	4	0	0.0	1.0	1.2	1.5	4	1.5	1.5
4.9	4	0	0.0	1.0	1.2	1.5	4	1.5	1.5
5.0	4	0	0.0	1.0	1.0	1.2	3	1.2	1.1

Table S10.3: Summary of the total length of runs $KB/1000$ on autosomes from the PLINK report (`--homozyg --homozyg-snp 25 --homozyg-kb hkb --homozyg-gap 100`) for variable minimum *ROH* length (Mb).

Mb	distinct	min	q1	med	mean	q3	max	iqr	sd
0.5	8	352.6	372.3	380.6	400.8	430.0	487.4	57.8	45.8
0.6	8	269.2	285.6	292.5	311.8	332.3	402.6	46.7	43.6
0.7	8	206.2	219.4	234.0	247.3	256.6	346.5	37.2	45.1
0.8	8	153.4	170.8	188.9	199.9	209.8	299.4	39.0	46.6
0.9	8	125.6	136.4	150.6	165.7	178.3	267.1	41.9	46.3
1.0	8	94.4	105.8	126.0	137.5	152.7	235.9	46.9	45.6
1.1	8	75.7	83.8	99.2	113.9	124.8	219.2	41.0	46.9
1.2	8	59.5	71.3	82.6	98.4	109.5	203.1	38.2	46.7
1.3	8	47.0	62.4	74.4	86.5	95.7	188.1	33.3	45.2
1.4	8	38.9	51.2	58.9	75.1	87.2	174.7	35.9	44.2
1.5	8	28.7	43.3	53.1	66.7	74.9	167.4	31.6	44.6
1.6	8	25.6	39.7	48.1	61.2	62.9	164.3	23.2	44.6
1.7	8	19.0	35.1	43.1	56.1	57.5	156.1	22.4	43.6
1.8	8	18.6	28.6	38.7	50.2	50.1	145.6	21.5	41.2
1.9	8	17.1	27.2	35.3	46.9	45.8	138.1	18.7	39.3
2.0	8	13.2	19.9	33.0	42.0	42.0	126.4	22.2	36.5
2.1	8	13.2	17.3	32.0	39.5	39.5	120.2	22.1	34.9
2.2	8	10.2	17.0	29.8	37.3	38.4	115.9	21.4	34.0
2.3	8	7.9	14.8	26.8	33.7	33.9	106.9	19.1	31.7
2.4	8	5.6	14.8	26.4	32.8	32.2	106.9	17.4	32.0
2.5	8	3.1	12.3	25.6	31.0	31.6	102.1	19.2	31.2
2.6	8	3.1	11.7	23.1	28.4	30.3	94.4	18.6	28.9
2.7	8	3.1	9.0	23.1	26.7	28.3	89.1	19.3	27.7
2.8	8	3.1	9.0	19.0	24.7	25.5	86.3	16.5	26.8
2.9	8	3.1	9.0	19.0	23.6	22.7	83.5	13.7	25.8
3.0	8	3.1	9.0	17.5	22.1	22.7	74.6	13.7	23.0
3.1	8	0.0	9.0	16.0	20.2	21.9	68.5	12.9	21.4
3.2	7	0.0	8.2	14.4	18.6	21.9	62.2	13.7	19.9

Mb	distinct	min	q1	med	mean	q3	max	iqr	sd
3.3	7	0.0	5.8	11.2	15.8	18.6	55.7	12.8	18.0
3.4	7	0.0	5.8	11.2	15.8	18.6	55.7	12.8	18.0
3.5	7	0.0	5.8	11.2	15.8	18.6	55.7	12.8	18.0
3.6	7	0.0	5.8	11.2	15.4	18.6	52.2	12.8	16.9
3.7	7	0.0	5.8	11.2	14.9	18.6	48.6	12.8	15.8
3.8	7	0.0	3.0	11.2	13.5	18.6	41.1	15.7	13.9
3.9	7	0.0	3.0	11.2	12.5	18.6	33.4	15.7	11.8
4.0	6	0.0	0.0	11.2	12.0	18.6	33.4	18.6	12.3
4.1	6	0.0	0.0	11.2	12.0	18.6	33.4	18.6	12.3
4.2	6	0.0	0.0	11.1	10.5	15.6	25.1	15.6	10.2
4.3	6	0.0	0.0	9.0	9.9	15.6	25.1	15.6	10.4
4.4	6	0.0	0.0	9.0	9.4	14.5	25.1	14.5	9.6
4.5	6	0.0	0.0	9.0	9.4	14.5	25.1	14.5	9.6
4.6	6	0.0	0.0	6.7	8.8	14.5	25.1	14.5	9.5
4.7	5	0.0	0.0	6.7	8.2	11.0	25.1	11.0	9.4
4.8	5	0.0	0.0	6.7	8.2	11.0	25.1	11.0	9.4
4.9	5	0.0	0.0	6.7	8.2	11.0	25.1	11.0	9.4
5.0	5	0.0	0.0	6.7	7.0	9.8	20.1	9.8	7.3

Table S10.4: Summary of the average length of runs $KBAVG/1000$ on autosomes from the PLINK report (`--homozyg --homozyg-snp 25 --homozyg-kb hkb --homozyg-gap 100`) for variable minimum ROH length (Mb).

Mb	distinct	min	q1	med	mean	q3	max	iqr	sd
0.5	8	0.77	0.79	0.80	0.83	0.84	0.98	0.04	0.07
0.6	8	0.89	0.91	0.94	0.97	0.99	1.19	0.08	0.10
0.7	8	1.00	1.04	1.08	1.11	1.14	1.37	0.11	0.12
0.8	8	1.13	1.16	1.22	1.26	1.28	1.58	0.11	0.15
0.9	8	1.23	1.29	1.36	1.39	1.42	1.76	0.13	0.17
1.0	8	1.33	1.44	1.51	1.54	1.56	1.98	0.12	0.20
1.1	8	1.47	1.60	1.66	1.71	1.78	2.13	0.18	0.22
1.2	8	1.58	1.72	1.82	1.86	1.94	2.28	0.22	0.23
1.3	8	1.73	1.81	1.96	1.99	2.08	2.44	0.28	0.25
1.4	8	1.83	1.96	2.12	2.16	2.39	2.61	0.44	0.28
1.5	8	2.01	2.10	2.30	2.32	2.53	2.70	0.43	0.26
1.6	8	2.08	2.18	2.47	2.44	2.72	2.74	0.54	0.28
1.7	8	2.16	2.35	2.61	2.58	2.78	3.00	0.44	0.29
1.8	8	2.32	2.43	2.86	2.74	2.98	3.08	0.55	0.31
1.9	8	2.32	2.49	3.06	2.85	3.10	3.18	0.61	0.36
2.0	8	2.32	2.84	3.18	3.03	3.27	3.40	0.43	0.38
2.1	8	2.42	2.94	3.23	3.12	3.39	3.54	0.44	0.41
2.2	8	2.54	2.94	3.35	3.21	3.48	3.76	0.54	0.44
2.3	8	2.64	3.09	3.51	3.38	3.72	3.90	0.63	0.48
2.4	8	2.73	3.11	3.51	3.45	3.77	4.08	0.66	0.51
2.5	8	2.82	3.33	3.56	3.55	3.90	4.08	0.58	0.45
2.6	8	2.93	3.33	3.62	3.69	4.15	4.43	0.83	0.57
2.7	8	3.07	3.40	3.76	3.80	4.33	4.43	0.93	0.54
2.8	8	3.07	3.54	3.84	3.96	4.64	4.79	1.10	0.67
2.9	8	3.07	3.54	3.87	4.06	4.83	5.07	1.30	0.79
3.0	8	3.07	3.54	3.95	4.16	5.00	5.40	1.46	0.89

Mb	distinct	min	q1	med	mean	q3	max	iqr	sd
3.1	8	0.00	3.54	4.11	3.86	5.14	5.40	1.60	1.76
3.2	7	0.00	2.74	4.33	3.52	5.14	5.40	2.40	2.25
3.3	7	0.00	2.91	4.67	3.91	5.72	6.48	2.82	2.54
3.4	7	0.00	2.91	4.67	3.91	5.72	6.48	2.82	2.54
3.5	7	0.00	2.91	4.67	3.91	5.72	6.48	2.82	2.54
3.6	7	0.00	2.91	4.73	3.92	5.72	6.48	2.82	2.55
3.7	7	0.00	2.91	4.78	3.93	5.72	6.48	2.82	2.55
3.8	7	0.00	2.98	4.92	3.98	5.72	6.48	2.74	2.57
3.9	7	0.00	2.98	5.14	4.03	5.72	6.48	2.74	2.60
4.0	6	0.00	0.00	5.14	3.54	5.72	6.48	5.72	2.97
4.1	6	0.00	0.00	5.14	3.54	5.72	6.48	5.72	2.97
4.2	6	0.00	0.00	5.29	3.72	6.31	6.48	6.31	3.13
4.3	6	0.00	0.00	5.52	3.78	6.31	6.48	6.31	3.15
4.4	6	0.00	0.00	5.72	3.84	6.40	6.48	6.40	3.21
4.5	6	0.00	0.00	5.72	3.84	6.40	6.48	6.40	3.21
4.6	6	0.00	0.00	5.72	4.07	6.41	8.28	6.41	3.48
4.7	5	0.00	0.00	5.72	4.30	6.86	8.28	6.86	3.71
4.8	5	0.00	0.00	5.72	4.30	6.86	8.28	6.86	3.71
4.9	5	0.00	0.00	5.72	4.30	6.86	8.28	6.86	3.71
5.0	5	0.00	0.00	5.93	4.44	7.41	8.28	7.41	3.81

Table S10.5: Summary of the inferred inbreeding F_{ROH} on autosomes from the PLINK report (`--homozyg --homozyg-snp 25 --homozyg-kb hkb --homozyg-gap 100`) for variable minimum *ROH* length (Mb).

Mb	distinct	min	q1	med	mean	q3	max	iqr	sd
0.5	8	0.122	0.129	0.132	0.139	0.149	0.169	0.020	0.016
0.6	8	0.093	0.099	0.102	0.108	0.115	0.140	0.016	0.015
0.7	8	0.072	0.076	0.081	0.086	0.089	0.120	0.013	0.016
0.8	8	0.053	0.059	0.066	0.069	0.073	0.104	0.014	0.016
0.9	8	0.044	0.047	0.052	0.058	0.062	0.093	0.015	0.016
1.0	8	0.033	0.037	0.044	0.048	0.053	0.082	0.016	0.016
1.1	8	0.026	0.029	0.034	0.040	0.043	0.076	0.014	0.016
1.2	8	0.021	0.025	0.029	0.034	0.038	0.071	0.013	0.016
1.3	8	0.016	0.022	0.026	0.030	0.033	0.065	0.012	0.016
1.4	8	0.014	0.018	0.020	0.026	0.030	0.061	0.012	0.015
1.5	8	0.010	0.015	0.018	0.023	0.026	0.058	0.011	0.015
1.6	8	0.009	0.014	0.017	0.021	0.022	0.057	0.008	0.015
1.7	8	0.007	0.012	0.015	0.019	0.020	0.054	0.008	0.015
1.8	8	0.006	0.010	0.013	0.017	0.017	0.051	0.007	0.014
1.9	8	0.006	0.009	0.012	0.016	0.016	0.048	0.006	0.014
2.0	8	0.005	0.007	0.011	0.015	0.015	0.044	0.008	0.013
2.1	8	0.005	0.006	0.011	0.014	0.014	0.042	0.008	0.012
2.2	8	0.004	0.006	0.010	0.013	0.013	0.040	0.007	0.012
2.3	8	0.003	0.005	0.009	0.012	0.012	0.037	0.007	0.011
2.4	8	0.002	0.005	0.009	0.011	0.011	0.037	0.006	0.011
2.5	8	0.001	0.004	0.009	0.011	0.011	0.035	0.007	0.011
2.6	8	0.001	0.004	0.008	0.010	0.011	0.033	0.006	0.010
2.7	8	0.001	0.003	0.008	0.009	0.010	0.031	0.007	0.010
2.8	8	0.001	0.003	0.007	0.009	0.009	0.030	0.006	0.009

Mb	distinct	min	q1	med	mean	q3	max	iqr	sd
2.9	8	0.001	0.003	0.007	0.008	0.008	0.029	0.005	0.009
3.0	8	0.001	0.003	0.006	0.008	0.008	0.026	0.005	0.008
3.1	8	0.000	0.003	0.006	0.007	0.008	0.024	0.004	0.007
3.2	7	0.000	0.003	0.005	0.006	0.008	0.022	0.005	0.007
3.3	7	0.000	0.002	0.004	0.005	0.006	0.019	0.004	0.006
3.4	7	0.000	0.002	0.004	0.005	0.006	0.019	0.004	0.006
3.5	7	0.000	0.002	0.004	0.005	0.006	0.019	0.004	0.006
3.6	7	0.000	0.002	0.004	0.005	0.006	0.018	0.004	0.006
3.7	7	0.000	0.002	0.004	0.005	0.006	0.017	0.004	0.005
3.8	7	0.000	0.001	0.004	0.005	0.006	0.014	0.005	0.005
3.9	7	0.000	0.001	0.004	0.004	0.006	0.012	0.005	0.004
4.0	6	0.000	0.000	0.004	0.004	0.006	0.012	0.006	0.004
4.1	6	0.000	0.000	0.004	0.004	0.006	0.012	0.006	0.004
4.2	6	0.000	0.000	0.004	0.004	0.005	0.009	0.005	0.004
4.3	6	0.000	0.000	0.003	0.003	0.005	0.009	0.005	0.004
4.4	6	0.000	0.000	0.003	0.003	0.005	0.009	0.005	0.003
4.5	6	0.000	0.000	0.003	0.003	0.005	0.009	0.005	0.003
4.6	6	0.000	0.000	0.002	0.003	0.005	0.009	0.005	0.003
4.7	5	0.000	0.000	0.002	0.003	0.004	0.009	0.004	0.003
4.8	5	0.000	0.000	0.002	0.003	0.004	0.009	0.004	0.003
4.9	5	0.000	0.000	0.002	0.003	0.004	0.009	0.004	0.003
5.0	5	0.000	0.000	0.002	0.002	0.003	0.007	0.003	0.003

Table S10.6: Summary of the inferred relatedness $R_{ROH} = 2xF_{ROH}$ on autosomes from the PLINK report (`--homozyg --homozyg-snp 25 --homozyg-kb hkb --homozyg-gap 100`) for variable minimum *ROH* length (Mb).

Mb	distinct	min	q1	med	mean	q3	max	iqr	sd
0.5	8	0.245	0.258	0.264	0.278	0.299	0.338	0.040	0.032
0.6	8	0.187	0.198	0.203	0.216	0.231	0.279	0.032	0.030
0.7	8	0.143	0.152	0.162	0.172	0.178	0.241	0.026	0.031
0.8	8	0.107	0.119	0.131	0.139	0.146	0.208	0.027	0.032
0.9	8	0.087	0.095	0.105	0.115	0.124	0.185	0.029	0.032
1.0	8	0.065	0.073	0.087	0.095	0.106	0.164	0.033	0.032
1.1	8	0.053	0.058	0.069	0.079	0.087	0.152	0.028	0.033
1.2	8	0.041	0.050	0.057	0.068	0.076	0.141	0.026	0.032
1.3	8	0.033	0.043	0.052	0.060	0.066	0.131	0.023	0.031
1.4	8	0.027	0.036	0.041	0.052	0.061	0.121	0.025	0.031
1.5	8	0.020	0.030	0.037	0.046	0.052	0.116	0.022	0.031
1.6	8	0.018	0.028	0.033	0.043	0.044	0.114	0.016	0.031
1.7	8	0.013	0.024	0.030	0.039	0.040	0.108	0.016	0.030
1.8	8	0.013	0.020	0.027	0.035	0.035	0.101	0.015	0.029
1.9	8	0.012	0.019	0.025	0.033	0.032	0.096	0.013	0.027
2.0	8	0.009	0.014	0.023	0.029	0.029	0.088	0.015	0.025
2.1	8	0.009	0.012	0.022	0.027	0.027	0.083	0.015	0.024
2.2	8	0.007	0.012	0.021	0.026	0.027	0.080	0.015	0.024
2.3	8	0.006	0.010	0.019	0.023	0.024	0.074	0.013	0.022
2.4	8	0.004	0.010	0.018	0.023	0.022	0.074	0.012	0.022
2.5	8	0.002	0.009	0.018	0.021	0.022	0.071	0.013	0.022
2.6	8	0.002	0.008	0.016	0.020	0.021	0.066	0.013	0.020

Mb	distinct	min	q1	med	mean	q3	max	iqr	sd
2.7	8	0.002	0.006	0.016	0.019	0.020	0.062	0.013	0.019
2.8	8	0.002	0.006	0.013	0.017	0.018	0.060	0.011	0.019
2.9	8	0.002	0.006	0.013	0.016	0.016	0.058	0.009	0.018
3.0	8	0.002	0.006	0.012	0.015	0.016	0.052	0.009	0.016
3.1	8	0.000	0.006	0.011	0.014	0.015	0.048	0.009	0.015
3.2	7	0.000	0.006	0.010	0.013	0.015	0.043	0.009	0.014
3.3	7	0.000	0.004	0.008	0.011	0.013	0.039	0.009	0.013
3.4	7	0.000	0.004	0.008	0.011	0.013	0.039	0.009	0.013
3.5	7	0.000	0.004	0.008	0.011	0.013	0.039	0.009	0.013
3.6	7	0.000	0.004	0.008	0.011	0.013	0.036	0.009	0.012
3.7	7	0.000	0.004	0.008	0.010	0.013	0.034	0.009	0.011
3.8	7	0.000	0.002	0.008	0.009	0.013	0.029	0.011	0.010
3.9	7	0.000	0.002	0.008	0.009	0.013	0.023	0.011	0.008
4.0	6	0.000	0.000	0.008	0.008	0.013	0.023	0.013	0.009
4.1	6	0.000	0.000	0.008	0.008	0.013	0.023	0.013	0.009
4.2	6	0.000	0.000	0.008	0.007	0.011	0.017	0.011	0.007
4.3	6	0.000	0.000	0.006	0.007	0.011	0.017	0.011	0.007
4.4	6	0.000	0.000	0.006	0.007	0.010	0.017	0.010	0.007
4.5	6	0.000	0.000	0.006	0.007	0.010	0.017	0.010	0.007
4.6	6	0.000	0.000	0.005	0.006	0.010	0.017	0.010	0.007
4.7	5	0.000	0.000	0.005	0.006	0.008	0.017	0.008	0.007
4.8	5	0.000	0.000	0.005	0.006	0.008	0.017	0.008	0.007
4.9	5	0.000	0.000	0.005	0.006	0.008	0.017	0.008	0.007
5.0	5	0.000	0.000	0.005	0.005	0.007	0.014	0.007	0.005

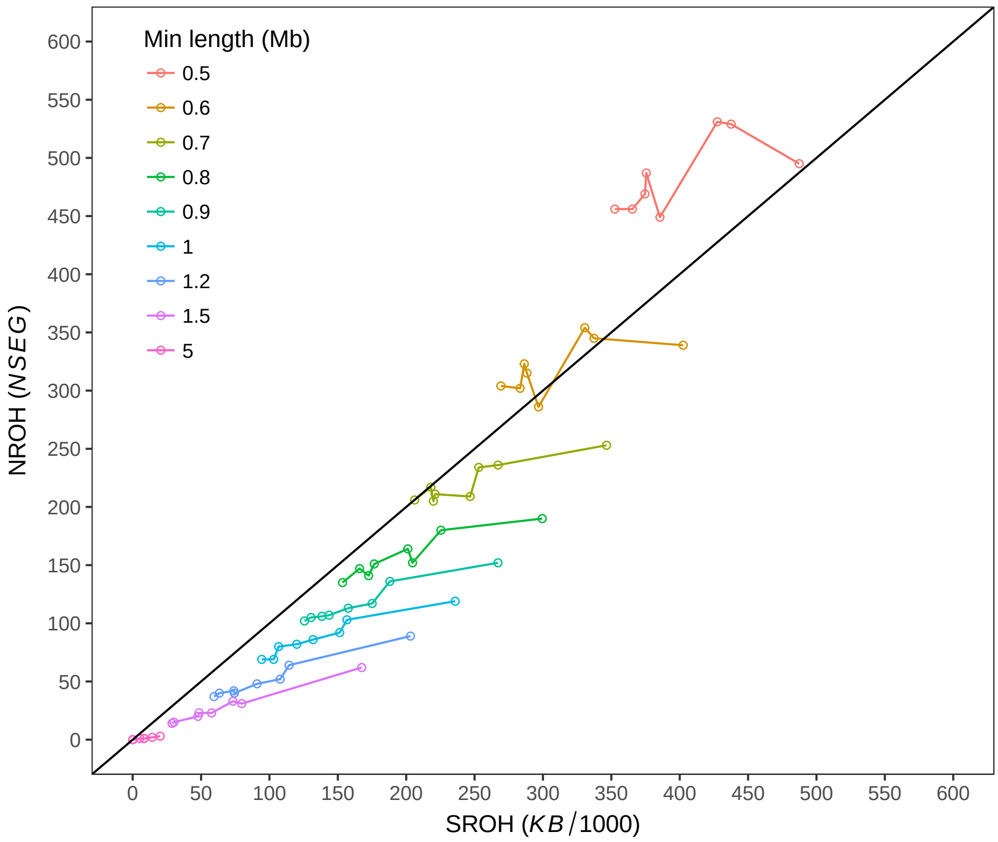

Figure S10.1: Plot of the number of runs of homozygosity NROH ($NSEQ$) against the total length of runs SROH ($KB/1000$) in the PLINK report for a subset $0.5-5$Mb of the variable minimum ROH lengths. At 0.6Mb minimum length the population seems bottlenecked with most points close to or at the diagonal. Above 0.6Mb the population seems bottlenecked and consanguineous with the points below and right shifted from the diagonal. At 1.5Mb the points left shift towards the diagonal, and at 5Mb the leftmost point is back near the diagonal.

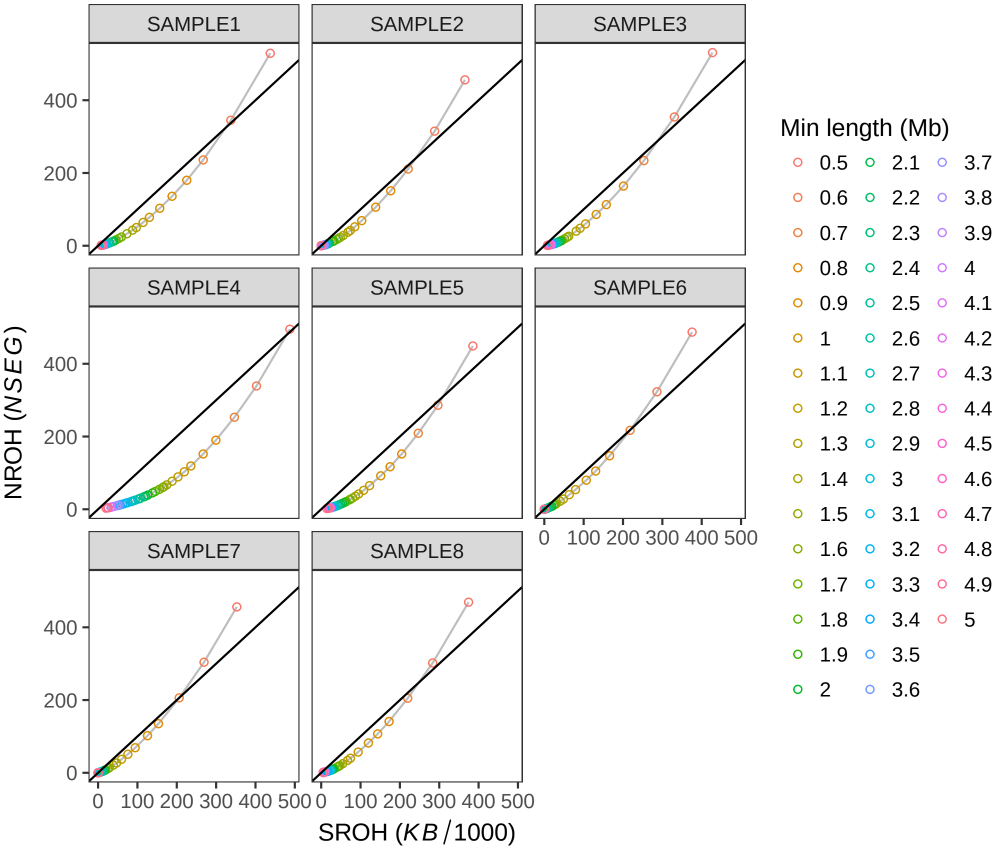

Figure S10.2: Plots of the number of runs of homozygosity NROH ($NSEQ$) against the total length of runs SROH ($KB/1000$) in the PLINK report for the minimum lengths of $ROH > 0.5$Mb. At about 1.0Mb the points are maximum shifted to the right from the diagonal.

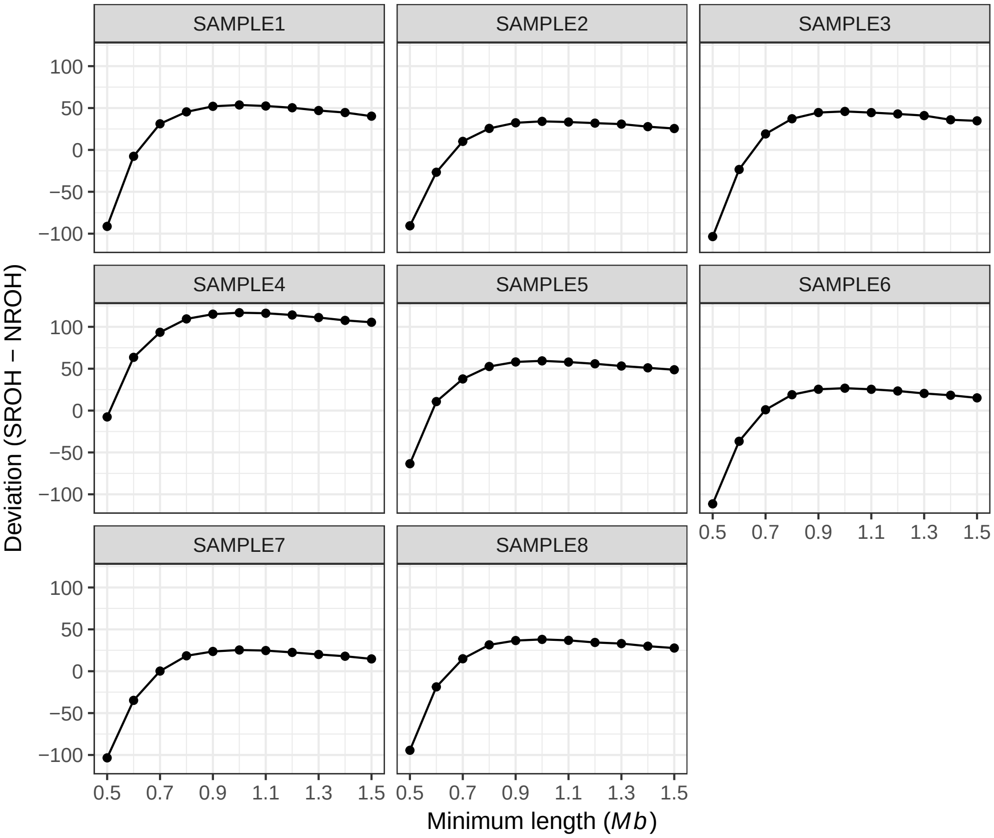

Figure S10.3: Plots of deviation SROH ($KB/1000$) − NROH ($NSEQ$) from the linear diagonal in the NROH versus SROH plots shown for minimum ROH lengths between 0.5 and 1.5Mb. For all the samples the SROH is below the diagonal (deviation > 0) for minimum ROH lengths above $0.7 - 0.8$Mb and the maximum deviation is found at 1.0Mb.

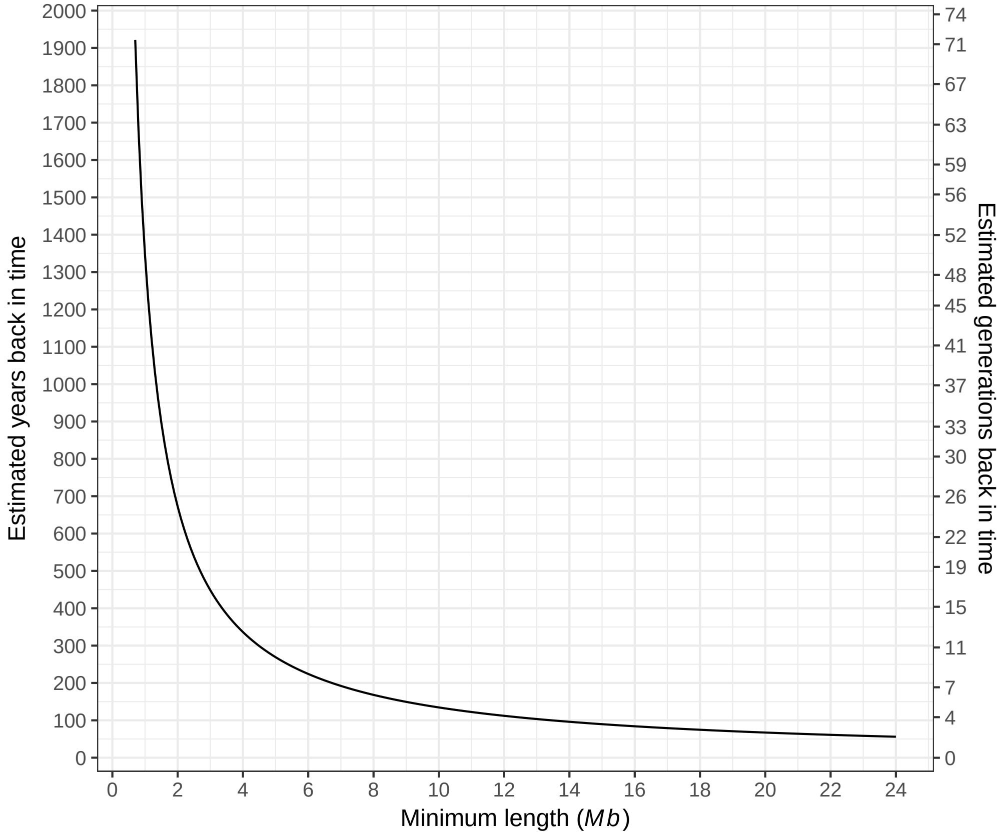

Figure S10.4: Plot of the estimated years back in time (left y-axis) and generations back in time (right y-axis) inferred from the minimum lengths of $ROH > 0.6$Mb. At 1Mb the estimate is 1345 years back and 50 generations back in time.

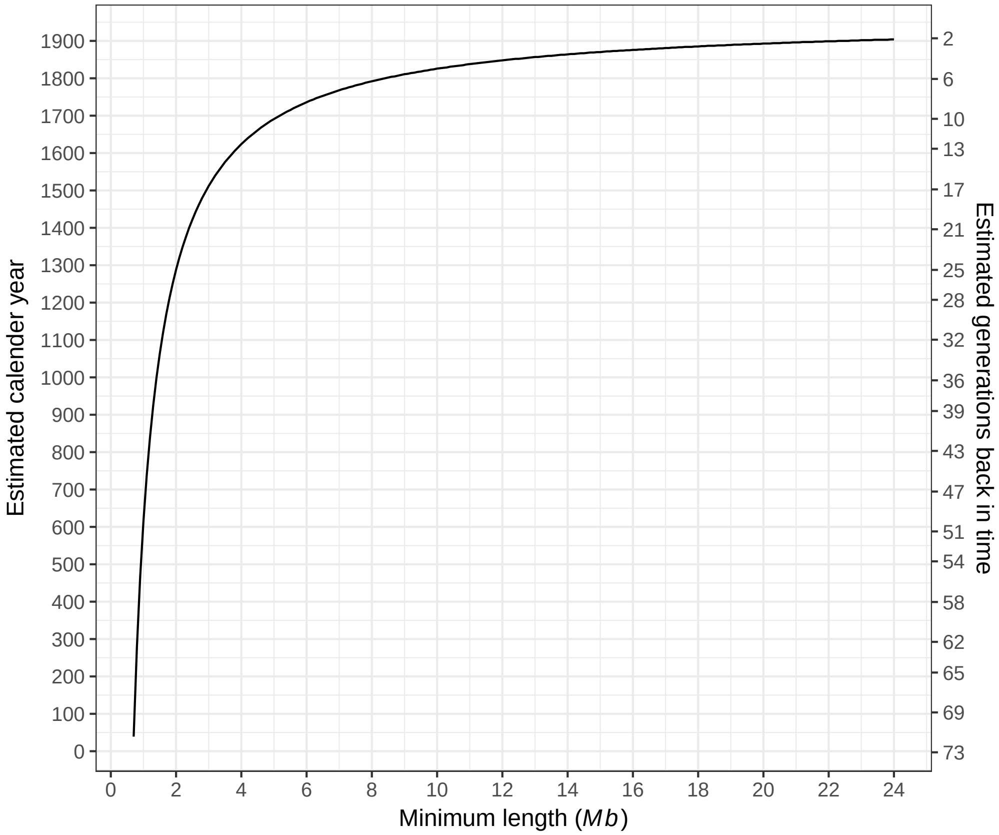

Figure S10.5: Plot of the estimated calender year (left y-axis) and generations back in time (right y-axis) since 1960 inferred from the minimum lengths of $ROH > 0.6$Mb. At 1Mb the estimated calender year is 615.

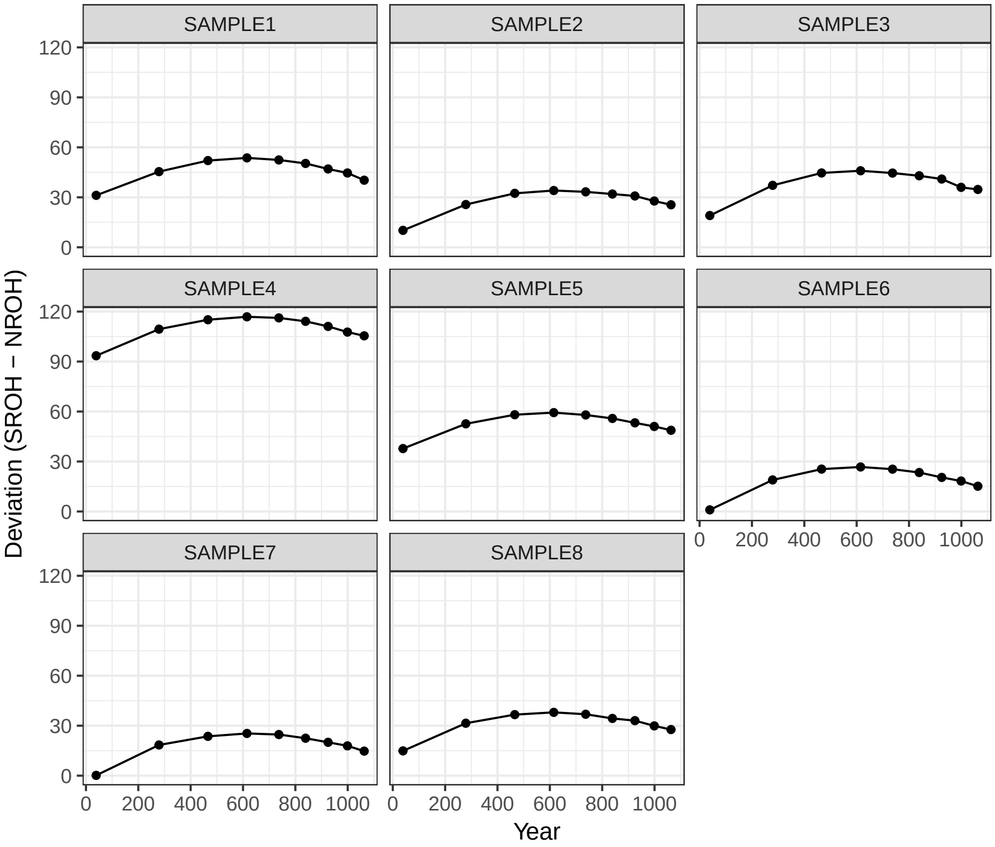

Figure S10.6: Plots of deviation SROH ($KB/1000$) $-$ NROH ($NSEQ$) from the linear diagonal in the NROH versus SROH plots shown for minimum ROH lengths between 0.7 and 1.5Mb, transformed to the corresponding estimated time in years. The maximum deviation for all samples is found at 1.0Mb corresponding to year 615.

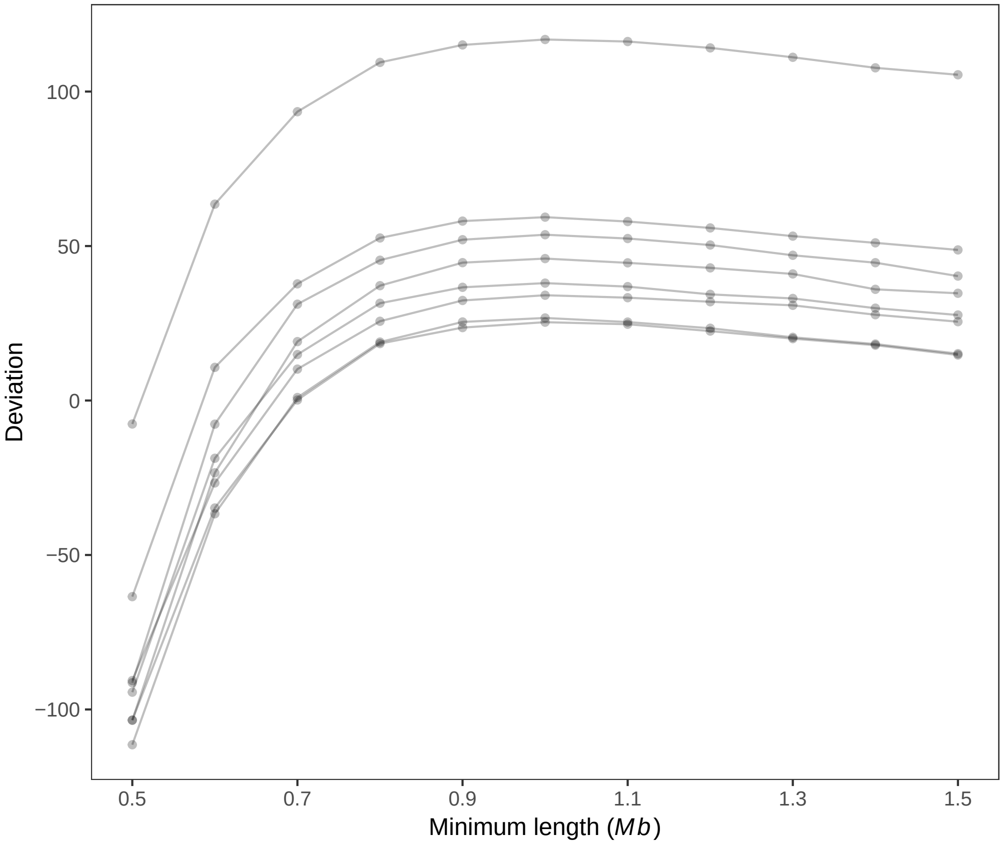

Figure S10.7: Plot of deviation SROH ($KB/1000$) − NROH ($NSEQ$) from the linear diagonal in the NROH versus SROH plots shown for minimum ROH lengths between 0.5 and 1.5Mb. For all the samples the SROH is clearly below the diagonal (deviation > 0) for minimum ROH lengths above $0.7 - 0.8$Mb and the maximum deviation is found at 1.0Mb.

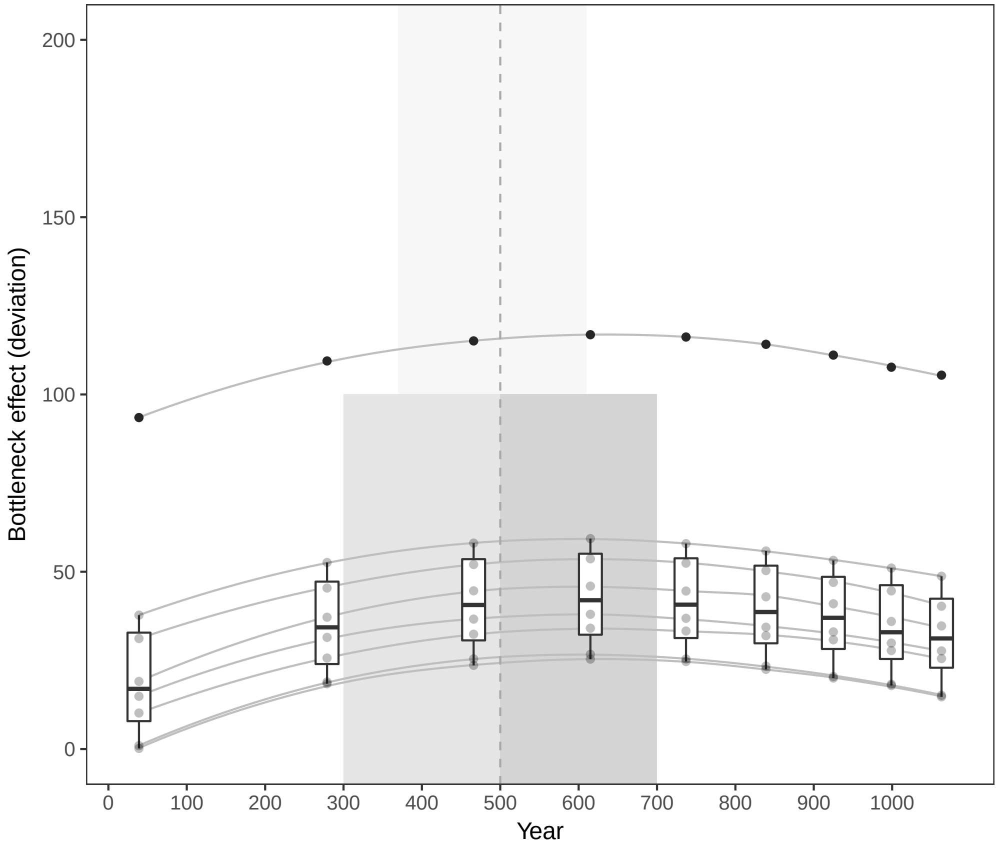

Figure S10.8: Plot of deviation from the linear diagonal in the NROH versus SROH plots shown for minimum ROH lengths between 0.7 and 1.5Mb, transformed to the corresponding estimated time in years. The maximum deviation for all samples is found at 1.0Mb corresponding to year 615. The three shaded rectangles illustrate the dating of the oldest archaeological findings from the Faroe Islands from two pre-Viking colonization events at year $300 - 500$ (left) and $500 - 700$ (right), and the first apperance of sheep DNA at year 500 (top, CI: $370 - 610$).
