# Supplementary material for: SNP heterozygosity, relatedness and inbreeding of whole genomes from the isolated population of the Faroe Islands
Source: BMC Genomics. 2023 Nov 23;24:707. doi: 10.1186/s12864-023-09763-x (PMC10666429; doi:10.1186/s12864-023-09763-x)
Supplement: Supplementary file 11 — Additional file 11. [file 12864_2023_9763_MOESM11_ESM.pdf]

Additional file 11 - Plot of ROH like Figure 1 for the advanced filter

Table S11.1: Filter (`bcftools filter -i`) used to filter the SNP files before further processing with PLINK.

Autosome files	Filter-text
SNP VCFs	FILTER='PASS' & QUAL>30 & FMT/DPU>10

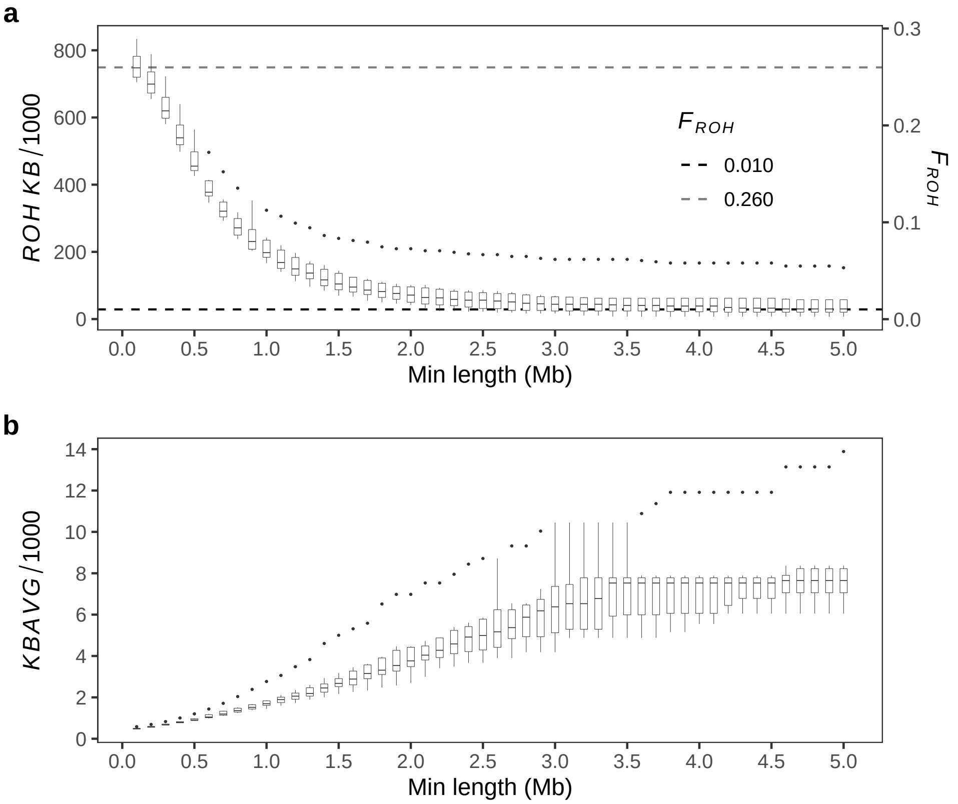

Figure S11.1: ROH of the eight merged genomes (autosomes) from the Faroe Islands. a, b Total $ROH = KB/1000$ and average $ROH = KBAVG/1000$ are the total and average length of ROH, respectively, in the PLINK –homozyg report for variable minimum segment length –homozyg-kb. This we varied from 100 to 5000kb in 100kb increments. a Boxplots of the total ROH (left axis) and of the inbreeding coefficient $F_{ROH} = KB/L$ (right axis) in which $L = 2881033.286$kb is the autosome length. The median inbreeding $F_{ROH>5} = 0.010$ (black dashed line) is the lowest level of recent inbreeding, which is like the average pedigree inbreeding of 0.0067 and 0.0081 estimated for multiple scelerosis patients and controls, respectively, from the Faroese population. The $F_{ROH>0.1} = 0.260$ (gray dashed line) is the highest level of both recent and ancient inbreeding, which is like $F_{ROH>0.1}$ for the European population of the 1000GP.
